# Technical Design Report for a Carbon-11 Treatment Facility

**DOI:** 10.3389/fmed.2021.697235

**Published:** 2022-04-25

**Authors:** Liviu Penescu, Thierry Stora, Simon Stegemann, Johanna Pitters, Elisa Fiorina, Ricardo Dos Santos Augusto, Claus Schmitzer, Fredrik Wenander, Katia Parodi, Alfredo Ferrari, Thomas E. Cocolios

**Affiliations:** ^1^Abstract Landscapes, Montpellier, France; ^2^European Organization for Nuclear Research (CERN), Geneva, Switzerland; ^3^Department of Physics and Astronomy, KU Leuven, Geel, Belgium; ^4^Istituto Nazionale di Fisica Nucleare (INFN), Sezione di Torino, Torino, Italy; ^5^Centro Nazionale di Adroterapia Oncologica (CNAO), Pavia, Italy; ^6^TRIUMF, Vancouver, BC, Canada; ^7^Ludwig Maximilian University of Munich (LMU), Munich, Germany; ^8^MedAustron, Wiener Neustadt, Austria

**Keywords:** particle therapy, carbon-11, radioactive isotopes, radioactive ion beams, particle accelerator, treatment planning

## Abstract

Particle therapy relies on the advantageous dose deposition which permits to highly conform the dose to the target and better spare the surrounding healthy tissues and organs at risk with respect to conventional radiotherapy. In the case of treatments with heavier ions (like carbon ions already clinically used), another advantage is the enhanced radiobiological effectiveness due to high linear energy transfer radiation. These particle therapy advantages are unfortunately not thoroughly exploited due to particle range uncertainties. The possibility to monitor the compliance between the ongoing and prescribed dose distribution is a crucial step toward new optimizations in treatment planning and adaptive therapy. The Positron Emission Tomography (PET) is an established quantitative 3D imaging technique for particle treatment verification and, among the isotopes used for PET imaging, the ^11^C has gained more attention from the scientific and clinical communities for its application as new radioactive projectile for particle therapy. This is an interesting option clinically because of an enhanced imaging potential, without dosimetry drawbacks; technically, because the stable isotope ^12^C is successfully already in use in clinics. The MEDICIS-Promed network led an initiative to study the possible technical solutions for the implementation of ^11^C radioisotopes in an accelerator-based particle therapy center. We present here the result of this study, consisting in a Technical Design Report for a ^11^C Treatment Facility. The clinical usefulness is reviewed based on existing experimental data, complemented by Monte Carlo simulations using the FLUKA code. The technical analysis starts from reviewing the layout and results of the facilities which produced ^11^C beams in the past, for testing purposes. It then focuses on the elaboration of the feasible upgrades of an existing ^12^C particle therapy center, to accommodate the production of ^11^C beams for therapy. The analysis covers the options to produce the ^11^C atoms in sufficient amounts (as required for therapy), to ionize them as required by the existing accelerator layouts, to accelerate and transport them to the irradiation rooms. The results of the analysis and the identified challenges define the possible implementation scenario and timeline.

## Introduction

Accelerators are used in a wide range of societal applications, the most notable being those related to external radiotherapy, and particularly with accelerated ion beams. When the first accelerators were developed, nuclear physicists realized soon after that they could trigger a new field of research *via* purified secondary Radioactive Ion Beams (RIB). This triggered the development and use of so-called Isotope mass Separation OnLine (ISOL) Facilities and Fragmentation facilities. A proof-of-concept application of the RIB to radiotherapy was performed at the Lawrence Berkeley National Laboratory, first at the Bevalac complex (1) and later under the BEARS collaboration, when it was demonstrated that a radioactive carbon ion, emitting positrons, could be used both for radiotherapy and imaging applications, exploiting the PET-imaging which was becoming a mature diagnosis imaging technique (2). Important developments further took place, with the first particle therapy facilities exploiting accelerated carbon ions coming online (based on the PIMMS design (3), as well as with new production and preparation techniques for isotope accelerators allowing the production of accelerated RIBs (notably implemented at REX-ISOLDE at CERN) (4).

The Marie-Curie training network MEDICIS-Promed brought together in a dedicated Work Package 15 young scientists across different institutes with Research Topics covering the chain from production to acceleration of ^11^C radionuclear beams (5). In strong contrast with stable ion beam facilities, the acceleration and delivery schemes of radioactive ion beams requires careful evaluation and optimized processes, because of the extremely limited quantities produced in the targets as opposed to large excess sources of stable ^1^H or ^12^C in case of stable beam facilities. Different production routes were investigated, and their suitability with the low energy preparation steps for injection in the Linac and subsequent acceleration schemes were investigated. Finally, the main scenarios to integrate the isotope production and acceleration into an existing hadron therapy facility were drafted.

## Motivation for Carbon-11 Beams: Overview and Modeling

The use of ^11^C for particle therapy can reduce the overall treatment time and increase the treatment quality (compared to the use of the stable isotope ^12^C). We detail in the present chapter how these improvements can be achieved, supported by simulation and experimental data.

Particle therapy relies on the advantageous dose deposition which permits to highly conform the dose to the target and better spare the surrounding healthy tissues and organs at risk (OAR) with respect to conventional radiotherapy (6). In the case of treatments with heavier ions (like carbon ions already clinically used and oxygen ions, planned for future clinical use), another advantage is the enhanced Relative Biological Effectiveness (RBE) due to high Linear Energy Transfer (LET) radiation.

These particle therapy advantages are not unfortunately thoroughly exploited due to particle range uncertainties. In fact, heavy charged particles show the characteristic dose distribution with a narrow Bragg Peak at the end of their range. In the most advanced beam delivery implementation of so-called pencil beam scanning, particle pencil beams have to deposit the dose distribution to the Clinical Target Volume (CTV) and Planning Target Volume (PTV) by precisely stopping into the patient body at the required depth. In literature (7), the range uncertainty contributions have been studied, identifying the sources of uncertainty both independent or dependent of dose calculation. Into the first category, there are beam reproducibility, patient positioning and setup, measurements in water for commissioning. In the latter group, there are CT calibration, tissue conversion, mean ionization energy estimation, range degradation for complex inhomogeneities.

In clinics, in order to design a robust treatment plan with respect to range uncertainties, safety margins of about (2.5–3.5)%+(1–3) mm (7) have to be considered during the treatment plan optimization procedure. In particular, this procedure aims at finding the most robust way to deliver the prescribed dose to the CTV and PTV minimizing the dose released in the Planning organ at Risk Volume (PRV) that represents the segmentation of the OAR with an additional margin related to position uncertainty.

Unfortunately, these safety margins are not enough to consider also patient's morphological changes that can occur during therapy (8, 9); such as tumor shrink/growth, inflammation, toxicity, loss of weight, cavities filling or emptying. Even though these variations are a well-known source of sub-optimal irradiation (10), they cannot be easily modeled or quantified because they strongly depend on the pathology and treated district.

To mitigate the unwanted degradation of dose distribution during the treatment course, patients who are affected by pathologies that are more prone to morphological changes, undergo periodic control Computed Tomography (CT) exams in order to check thanks to the Treatment Planning System (TPS) calculation that the actual delivered dose on the new patient morphology is still compliant with the prescription to the CTV and adequate for OARs limits. If necessary, these control CTs can be used to replan the treatment. For example, in (11) a retrospective study over 730 patients, affected by cranial and extracranial tumor, shows that an adaptive replanning was required in 5.5% of cases due to morphological or anatomic changes.

The possibility to monitor the compliance between the ongoing and prescribed dose distribution is a crucial step toward new optimizations in treatment planning and adaptive therapy. Therefore, in the last decades, *in vivo* treatment verification devices, based on the detection of secondary radiation, have been explored. They detect the products of the nuclear interactions between the primary beam and patient tissues, such as prompt photons obtained from nuclear de-excitation, secondary charged particles generated by nuclear fragmentation, and annihilation photons coming from positron emitters (12–14). Among them, Positron Emission Tomography (PET) is an established quantitative 3D imaging technique for particle treatment verification. The annihilation signal can be acquired both during and after the irradiation and presents a very good correlation to Bragg peak for heavy ions such as carbon or oxygen due to projectile fragmentation and related positron emitter production.

In the case of proton and carbon ion particle therapy, oxygen and carbon positron emitters are the most abundant products and their half-life is of the order of minutes or seconds. In particular, ^11^C has an half life of 20 min and the distribution of the ^11^C isotopes induced during ^12^C ion irradiation shows a peak well correlated with the Bragg Peak position because projectile fragmentation (15). In the case of ^12^C irradiation, the production of ^11^C has a small cross section; in total about 2% of the primary carbon ions undergo nuclear reactions for each cm of range in water (16). As a consequence, only about few percent of the primary ^12^C projectiles have been fragmented in ^11^C, yielding a PET image that is noisy and may require, depending on the detection efficiency, acquisition strategy and (for in-beam implementations) accelerator duty cycle, long acquisition time with respect to the delivery time to be significant.

Three different workflows for implementing treatment verification by means of a PET device have been explored (17, 18): off-line (PET/CT), in-room (PET or PET/CT) and in-beam (PET).

Off-line PET/CT relies on a commercial full-ring scanner sited outside the treatment room. The integrated CT system is useful for PET image co-registration on the planning CT. This instrumentation has a comparably low costs and PET images have good quality due to the full ring geometry. Nevertheless, the effectiveness of treatment verification is limited by the biological wash-out and the limited counting statistics due to the short decay time of the positron emitters along with positional uncertainties due to the patient repositioning. The clinical workflow into the treatment room is not slowed down with respect to the normal clinical routine. However, the off-line PET image requires long acquisition time for accumulating sufficient counting statistics [up to 30 min (19)] and this aspect has an indirect impact on the clinical routine and requires additional personnel.

In-room PET is based on a stand-alone full-ring PET or PET/CT scanner positioned inside the treatment room. With this configuration, the biological wash-out and the corresponding signal degradation are mitigated and a state-of-the-art PET image can be obtained in a reduced acquisition time with respect to the off-line PET [about 5 min (17)]. In order to minimize the patient repositioning uncertainty, in some in-room solutions, the same treatment couch can be also used. The main drawbacks are the slowing down of the clinical workflow in the treatment room and the need of radiation hard technology.

In-beam PET exploits a custom PET detector, able to acquire data during patient irradiation. In this operational modality, several geometrical constraints must be addressed for compatibility with the beam line and the clinical procedures and, therefore, a dual-head geometry (15, 20–22) or a complex full-ring geometry (23) have been investigated. The in-beam PET approach is the only solution to online verify the compliance of the ongoing and prescribed treatment. Biological wash-out, signal degradation and patient positioning uncertainty are strongly reduced but, on the other hand, since the in-beam PET devices are prototypes, there are high integration costs in the clinical routine.

The PET-based treatment verification can be performed in two ways. First, an inter-fractional comparison can be made by considering the experimental PET images of consecutive days or with respect to the PET image acquired in the first session of therapy (24). This approach relies on the reproducibility of the measurement. Another treatment verification approach is based on Monte Carlo simulations and aims at evaluating both accuracy and reproducibility of the experimental measurement (25). Moreover, some studies (26–29) investigated the possibility to analytically calculate the distribution of the positron emitters from planned dose information and, recently, these fast analytical approaches have been implemented into research Treatment Planning Systems (TPS) and compared with Monte Carlo simulations (27, 30).

In literature, several strategies and algorithms for treatment quality verification by means of PET images have been developed. Most of them rely on the identification of the activity distal fall-off with quantitative and automated methods [e.g., (24, 28, 31)] or visual analysis (32).

The main isotopes important in PET imaging verification in particle therapy are ^11^C, ^10^C and ^15^O. They are characterized by a relatively short half-life: 20 min for ^11^C, 20 s for ^10^C and 2 min for ^15^O.

Among them, the ^11^C has gained more attention from the scientific and clinical communities for its application as new radioactive projectile for particle therapy. This interest has been driven by its advantageous RBE with respect to protons and its reduced fragmentation with respect to oxygen (33). Moreover, the stable isotope ^12^C is successfully already in use in clinics. By comparison, the ^10^C would give a more prompt signal on the beam position but the very short decay time will lead to problems during acceleration to avoid a reduced statistics. ^11^C distribution can be acquired for minutes also after the irradiation although, in principle, the PET image will be affected by wash-out. Anyway, in the case of radioactive ^11^C beams, almost all the projectiles become useful probes for treatment verification and therefore the gain in statistics will lead to shorten the acquisition time and mitigate this drawback.

Comparing with its stable counterpart, ^11^C has the potential of improving PET signal counts by over a factor of 10 in offline PET acquisition mode and up to a factor of two in online mode, at the respective distribution's peak. Notwithstanding that, the signal peak resulting from ^11^C originates directly from the beam particles whereas the signal from stable carbon ion irradiation proceeds from positron emitters produced *via* fragmentation reactions. Consequently, the peak of the signal arising from the ^11^C irradiation tends to be better correlated with the Spread-Out Bragg Peak (SOBP) leading peak range, unlike the ^12^C case. Even though the effect is less evident in the online acquisition mode, due to relatively long half-life of ^11^C (~20 min) its use still allows for an easier identification of the SOBP range, overcoming the neutron-induced background, provided a reverse SOBP energy layer order is employed. Thus, this effect can lead to a more straightforward evaluation of the absorbed dose distribution and could have positive impact in range and treatment verification using in-beam PET techniques.

Experimental data pertaining ^11^C dosimetry and PET imaging performance have been obtained at QST/NIRS-HIMAC in Chiba, Japan. The experimental data consisted of: Bragg peak curves for stable and radioactive carbon ion beams in water; PET scanning and image acquisition, in between synchroton accelerated ion beam delivery (inter-spills) and continuing afterwards, for stable and radioactive carbon ions in PMMA. These data were then subsequently used to benchmark FLUKA code predictions (34). The ^11^C ion beam was generated *via* an in-flight fragmentation method in HIMAC's secondary beam course, exploiting the interaction of the synchrotron accelerated main (^12^C ion) beam with a beryllium target (35–40).

Although this method achieves production rates of almost 1%, which are deemed sufficient for testing purposes, the radioactive ion beams produced are considerably broad and feature larger momentum spreads than the projectile beam (36, 38). Moreover, the production method is also characterized by the presence of impurities in the secondary beam, originating from the projectile fragments. In the presented case, the impurity level reached about 7% (34).

To support this approach and to allow detailed analysis, a Monte Carlo code simulation data can provide a valuable insight into carbon ion hadrontherapy treatment planning, verification, optimization and eventually its outcome (25, 41–47). Recent developments in the FLUKA code have enhanced the accuracy of the models governing ion transport and interactions, resulting in an improved reproduction of the fragmentation mechanisms and thus a more reliable dosimetry and imaging estimate (48–50). Furthermore, the recently developed FLUKA PET tools enable the simulation of a PET scanner performance as well as signal acquisition throughout and after the irradiation, providing a more direct assessment of the imaging gain (51, 52).

A recent example of image performance evaluation used a SIEMENS Biograph mCT PET scanner from Heidelberg Ion Therapy Center as model, as well as a synchrotron-like irradiation with either ^11^C or ^12^C ions, simulating SOBP of comparable dose and range delivered to an antropomorphic head voxelized structure (52).

All the above-mentioned factors support the enhanced ^11^C ion irradiation imaging potential, without dosimetry drawbacks. Furthermore, in studies carried out by QST/NIRS and ANSTO (33), the relative biological effectiveness of radioactive ion beams of ^11^C (and ^10^C) has been found to be equivalent to that of their stable counterpart. Moreover, the same study corroborates the higher positron emitter production and comparable dosimetry performance of ^11^C ions with respect to stable carbon ions.

Also, encouraging results to treat tumors in small animals with radioactive ion beams have been obtained recently at the BARB Experiment at GSI, in the framework of the Super-FRS collaboration (53). The further proof-of-concept will focus on the application of ^11^C and ^15^O.

## Production of ^11^C Beams: Overview of Past Results

The possible advantages of using radioactive ion beams for PET-aided hadron therapy already have a long history (54–56). Worldwide, several facilities have attempted to produce ^11^C beams using different techniques:

- Lawrence Berkeley National Laboratory
- Center de Recherche du Cyclotron
- GANIL
- CERN ISOLDE
- ISAC/TRIUMF
- HIMAC/NIRS.

This chapter aims to provide an overview of how those facilities produced ^11^C beams, the technical details and the beam properties and particularities, as well as the developments with respect to high intensity ^11^C beam production. To get a broader picture of the past ^11^C experiments and future related perspectives, the reader is directed also to a recently-published overview focused on the medical use of the ^11^C beams produced to this day (57).

### Lawrence Berkeley National Laboratory

The Bevalac at the Lawrence Berkeley National Laboratory facility was an accelerator complex, established in 1974 when coupling the SuperHILAC linear accelerator (8.5 MeV/u) and the Bevatron proton synchrotron of 6.2 GeV energy (58, 59). The Bevalac was used for the production of heavy ion beams for both research and radiation therapy and is credited as one of the pioneering facilities for accelerated radioactive ion beams (60). Between 1977 and 1992, 433 patients were treated, where most of the treatments were performed with a 670 MeV/u neon beam (61). Before its decommission in 1993, ^11^C beams were produced by projectile fragmentation using different initial beams and thin targets. A beam of 2 x 10^7^ ions per pulse was produced by bombarding a 7.8 cm thick Be target with a primary 1.5 x 10^10^ ions per pulse ^12^C beam with an average energy of 350 MeV/u. This corresponds to a total efficiency of 1/750 ions per primary. It was reported that the primary beam was expected to suffer from 100 MeV/u energy loss after reaction, yielding a ^11^C beam with angular spread of approximately ±10 mrad and a momentum spread of ±1% (increased to ±12 mrad and ±2% when considering multiple scattering in the target). It was further reported that an excellent separation from the primary beam was achieved using a magnet with 1/500 resolving power (1). Besides that, a ^11^C beam was produced by bombarding a 1” (2.5 cm) Be target with ^18^O beam of 800 MeV/u energy, and the production cross section of ^11^C from a 375 MeV/u Ne^10+^ beam hitting in a polystyrene target consisting of two disks with 3” (7.6 cm) diameter and 0.25” (0.64 cm) thickness was measured (62, 63).

In 1998, the BEARS initiative was launched at Lawrence Berkeley National Laboratory, which aimed to expand the RIB capability (64). For this purpose, a 350 m transfer line was built between the 11 MeV PET-cyclotron at the Biomedical Isotope Facility (BIF) and the 88” (~224 cm) cyclotron of the Nuclear Science Division. ^11^C was produced irradiating for 5 min a 13 ml (80 mm deep) N_2_ gas target (filled to 22 atm) with 10 MeV protons and ~30 μA intensity of the medical cyclotron (2). 0.2% O_2_ was mixed into the gas target to produce ^11^CO_2_ to allow gaseous transport and cryogenic separation. The concentration of O_2_ was chosen to have sufficient oxygen available for ^11^CO_2_ formation, while avoiding overloading the ion source with non-radioactive chemical species formed during irradiation. The gas mixture was transported via a capillary system to the cryogenic trap for separation and subsequent injection into the AECR-U ECR ion source. It was found that the cryogenic trap was a crucial feature for the performance of the ion source. The AECR-U is a two-frequency (14 and 10 GHz) ECR ion source that provided an ionization efficiency distribution (by ion charge states) of: 3+ = 4%, 4+ = 11%, 5+ = 4%, 6+ = 2% (2). The ion source was operated at pressures of the order of 1 x 10^−7^ Torr (~1 x 10^−7^ mbar), and the 6+ charge state was selected using a stripper foil to erase boron contaminations. The entire system was operated by a fully automatized control system, handling the loading of the target, the irradiation and the unloading. Using a 5 min cycle, a final beam intensity of 1 x 10^8^ ions/s with an energy of 120 MeV was achieved (2).

### Center de Recherche du Cyclotron

The RIB facility at the Center de Recherche du Cyclotron (CRC) in Louvain-la-Neuve, established in 1989 and in operation until 2009, was the first facility that coupled an ISOL-type RIB production system to a post-accelerator, therefore, providing the first post-accelerated RIBs (65, 66). The accelerator complex comprised three accelerators: CYCLONE30 is a 30 MeV proton accelerator with beam intensities up to 300 μA developed for medical purposes (67), while both CYCLONE44 (K = 40) and CYCLONE110 (K = 110) are cyclotrons for the post-acceleration of nuclei produced with CYCLONE30. Two types of ion sources were in operation: firstly, sputtering ion sources, consisting of a biased electrode containing the material to ionize (67); secondly, a 6 GHz ECR ion source was developed for fast, low charge state ionization. Offline measurements using calibrated CO_2_ leaks yielded a 15% ionization efficiency for C^+^, at an in-source pressure of 1 x 10^−5^ mbar (67). For the production of ^11^C, two boron based powder targets were tested, boron nitride (BN) and boron oxide (B_2_O_3_) (68, 69). For both materials, a release study was performed. B_2_O_3_ melts at 450°C but vitrifies to a glass-like substance after cooling. With the initial melting, a strong outgassing and the formation of bubbles was observed, causing the material to expand. However, once vitrified, the material showed normal melting behavior. Hence, prior to irradiation, the material was vitrified by carefully pre-heating to 800°C and cooling subsequently. The release study of B_2_O_3_ showed a higher release efficiency at lower temperatures. However, only one cycle could be observed due to the escaping of the powder from the target container. In the case of BN, 6 g of powder was compressed to 0.8 g/cm^3^ into a graphite cavity and outgassed prior to irradiation. The release of ^11^C was rather limited at lower temperatures, however an efficiency of 10% could be obtained at 1,000°C in several different runs. It was observed that this characteristic resulted from the lack of free O_2_ available for the formation of carbon oxides. Therefore, an oxygen leak was added providing a partial pressure of approximately 1 x 10^−2^ mbar. No improvement was observed, which was probably due to an unpractical placement of the O_2_ leak and the oxygen strongly reacting with the surrounding carbon of the hot graphite cavity. It was later reported elsewhere that an on-line experiment using a BN target operated with an oxygen leak of 0.1 cm^3^/h resulted in a ^11^C beam of 1 x 10^7^ ions/s (70).

### CERN ISOLDE

Since 50 years, CERN ISOLDE (Isotope Separator On Line DEvice) (71) produces various radioactive ion beams from the chart of nuclides. ISOLDE receives 1.4 GeV protons from the Proton Synchrotron Booster (PSB) of the CERN accelerator chain with intensities up to 2 μA. Three different types of ion sources are available for 1+ charge state ionization: surface ion sources, plasma ion sources and laser ion sources.

At ISOLDE, many target-ion source combinations have been developed over the years, allowing to produce radioisotopes from more than 74 different elements. For mass separation, two separators are available that are operated with independent target-ion source units. The General Purpose Separator (GPS), equipped with one bending magnet and an electrostatic switchyard, allows to extract three mass separated beams simultaneously. For higher resolving power (>5,000), the High Resolution Separator (HRS) is available, consisting of two bending magnets. The experimental hall of ISOLDE hosts many different experiments that can receive the beam from either GPS or HRS. Several mass separated ^11^C beams have been produced from different target-ion source units, as can be seen in Table 1. It is remarkable that almost all beams were observed in the CO^+^ sideband. Furthermore, the oxide targets showed that CO^+^ was exceeding CO_2_ by a factor of 10 to 100 (75), and for the NaF:LiF molten salt target, providing so far the highest yield, CO^+^ was 30 times stronger than CO_2_ (74).

**Table 1:** ^11^C beams produced at CERN-ISOLDE.

**Target**	**Yield [1/μC]**	**Ion source**	**Molecular sideband**	**References**
HfO_2_ fibers	4.4 x 10^4^	Plasma-Helicon		(72)
TiO_x_ fibers	6.2 x 10^6^	Plasma-Cold-MK7	C^16^O^+^	(73)
NaF:LiF salt	7.7 x 10^8^	Plasma-Cold-VD7	^11^C^16^O^+^	(74)
MgO	2.1 x 10^5^	Plasma-Cold-MK7	C^16^O^+^	(73)
CeO_x_ fibers	4.8 x 10^6^	Plasma-Cold-MK7	C^16^O^+^	(73)
CaO nanostructured powder	2.7 x 10^6^	Plasma-Helicon	^11^C^16^O^+^	(72)

Besides that, extensive research has been performed for the production and extraction of short-lived carbon beams (75, 76). Adsorption enthalpies of CO and CO_2_ have been measured for several materials: MgO, Al_2_O_3_, SiO_2_, CaO, TiO_2_, ZrO_2_, HfO_2_ and Y_2_O_3_. High adsorption enthalpies result in longer retention/sticking times of CO or CO_2_ on such surfaces, and therefore, reduce the yields. SiO_2_ and Al_2_O_3_ were investigated as coating materials for transfer lines and ion sources. It was found that for CO the retention times are for both materials negligible, but for CO_2_ retention on Al_2_O_3_ becomes more evident at temperatures below 400°C (76). Diffusion studies of ^11^C in MgO, TiO_2_ and HfO_2_ as pressed powder and pressed fiber pellets showed that diffusion in fiber pellets is faster than in pressed powder pellets. It was furthermore concluded that limitations on the extraction and transport of short-lived carbon isotopes as carbon oxides mainly result from a shortage of oxygen supply, losses on hot tantalum surfaces (>1,000°C) of the target unit and retention due to the adsorption on hot molybdenum surfaces in the ion source delaying the extraction (75).

### SPIRAL1/GANIL

Since 2001 SPIRAL1 at GANIL produces radioactive ion beams via the ISOL method (77). The facility hosts five different cyclotrons for the production and acceleration of RIBs (78). The two low-energy cyclotrons C01 and C02 send beams to the irradiation beam line IRRSUD (<1 MeV/u). CSS1 (4 to 13 MeV/u) and CSS2 in series post-accelerate stable beams up to 95 MeV/u, which are then send on a graphite target for radioisotope production. The produced radioisotopes diffuse to a Nanogan-3 ECR ion source for multi-charge ionization (41). After mass separation (250 resolving power), the beam can either be send into the low-energy beam line LIRAT or can be injected into the CIME cyclotron (K = 265) for post-acceleration (1.7 to 25 MeV/u). Two projects are currently ongoing aiming to expand GANIL's RIB inventory:

- Firstly, the SPIRAL1 upgrade is being finalized, containing new target-ion source systems for more 1+ RIBs. Furthermore, a Phoenix type charge booster is being installed for 1+ to n+ charge breeding (77).
- Secondly, the SPIRAL2 project will provide beams produced via the ISOL and the in-flight technique.

Although, no ^11^C beam has been produced yet at SPIRAL1, studies on CO and CO_2_ ionization and charge breeding efficiency have been performed (76, 79). A 2.45 GHz ECR ion source for efficient 1+ ionization was developed at GANIL (MONO 1000). Based on this design, a compact version was developed and tested in an off-line study at ISOLDE and measured an ionization efficiency of 14% for CO^+^.

### ISAC/TRIUMF

TRIUMF in Vancouver (Canada) is a national laboratory for nuclear and particle physics. Their main accelerator is a sector-focused cyclotron with four independent beam extraction lines that accelerates H^−^ ions with a total beam current of 300 μA to energies ranging from 70 to 520 MeV (80). One of these extraction lines enters the ISAC facility, providing proton beams of 500 MeV with up to 100 μA beam intensity for radioactive ion beam production. ISAC comprises one target station with three types of ion sources: surface ion source, resonant laser ion source, plasma ion source (FEBIAD). A separator consisting of two magnets in series separates the ions extracted from the ion source. Currently, a new laboratory is under construction, which will add two more target stations to the inventory of ISAC. In detail, this is the Advanced Rare IsotopE Laboratory (ARIEL) project, which will add another 500 MeV, 100 μA proton beam line and a 50 MeV, 500 kW electron beam line (80).

Up to now, many radioactive ion beams have been produced from numerous targets (81). For the production of radioactive ^11^C beams, a composite NiO/Ni target was developed and tested on-line in 2012 and 2013 (82). The target was operated at a maximum temperature of 1,100°C to prevent high vapor pressures overloading the FEBIAD ion source, which would reduce the ionization efficiency. A high-power target container was used to dissipate the deposited beam power of the 500 MeV proton beam with a maximum intensity of 16 μA. Throughout several runs, a maximum ^11^CO^+^ yield of 1 x 10^7^ ions/s was observed. It is worth mentioning that a ratio CO^+^ to C^+^ of ~10 was observed, where the C^+^ beam is presumably originating from molecular breakup in the ion source (80).

### HIMAC/NIRS

Since the National Institute for Radiological Sciences (NIRS) completed in 1994 the construction of the Heavy Ion Medical Accelerator (HIMAC) in Chiba (Japan), >10,000 cancer patients have been treated using high-energy carbon beams (83). The original accelerator complex consisted of three ion sources, an RFQ cavity, an Alvarez type Drift-Tube-Linac, a pair of synchrotron rings and beam transport lines. The HIMAC can accelerate heavy ions from protons to xenon up to 800 MeV/u for a charge-over-mass ratio >0.5 (83). In 2010 a new treatment facility was added next to HIMAC, comprising a superconducting rotating-gantry and 3D raster-scanning irradiation techniques. NIRS has a strong R&D programme since 2004 and designed a compact accelerator facility for more cost-effective and size-reduced treatment centers.

At first, ^11^C beam production was studied using the in-flight projectile fragmentation technique (37). ^11^C was obtained by sending a primary 430 MeV/u ^12^C beam onto a Be target. A set of two bending magnets was used for separation and final focusing was achieved by a triplet of quadrupole magnets. This study showed that the yield strongly depends on the target thickness, degrader thickness (when used) and the angular acceptance. Remarkable is that increasing the degrader thickness from 0 to 10.6 mm, the beam purity is increased from 93 to 99%, however, decreasing the yield from 0.97 to 0.76%. For most of the tests, no degrader was used, resulting in a relatively poor beam purity of 93% with contaminations of ^12^C and ^7^Be. It was pointed out that these yield dependencies made the end cut of the depth dose distribution vague and not desirable (37). Finally, using a 1.8 x 10^9^ pps ^12^C beam, 7.2 x 10^6^ pps of ^11^C were delivered using spot scanning, which is insufficient in respect of dose delivery. Furthermore, large momentum spread and emittance resulted in undesirable beam characteristics.

**Figure 1:**
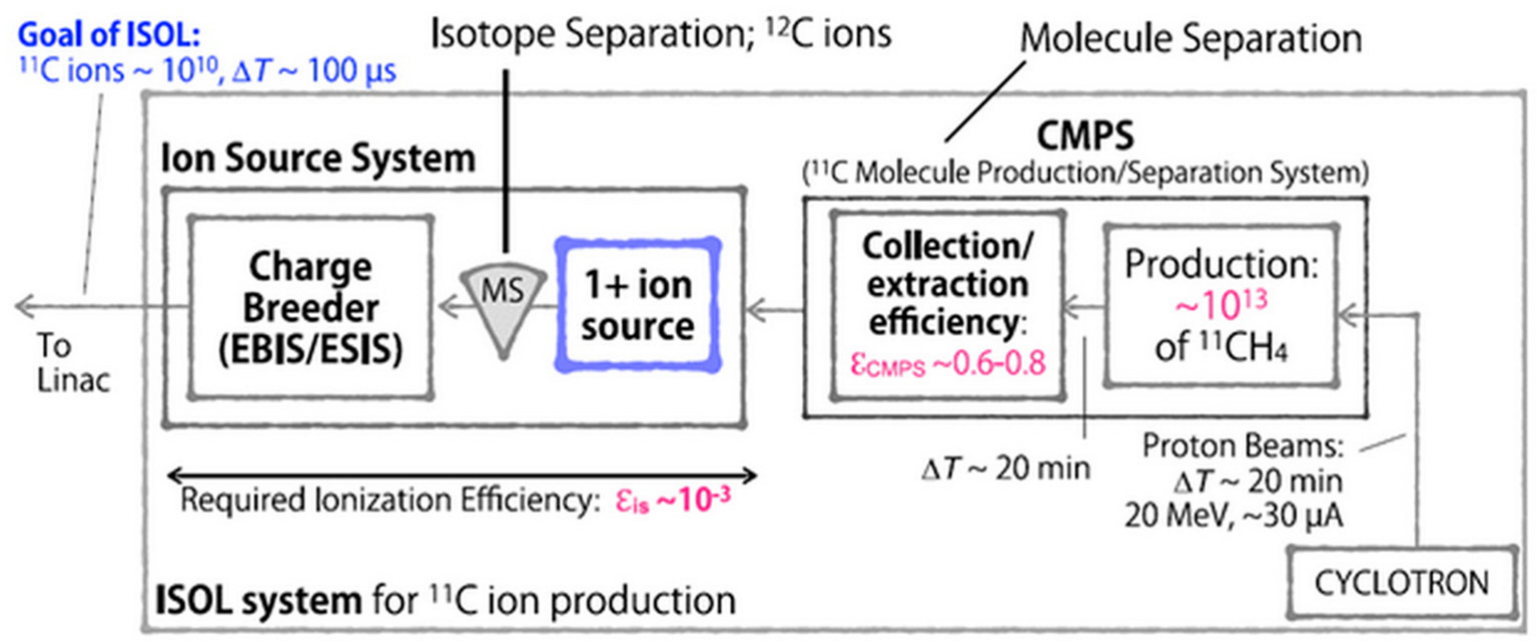
Possible ISOL-type ^11^C beam production system, proposed by NIRS (84).

More recently, studies on producing ^11^C beams using the PET-isotope production scheme from N_2_ gas targets and using the ISOL method were performed (85, 86). The first route, *via* the ^14^N(p,α)^11^C reaction from N_2_ gas targets, was a theoretical calculation starting with an 18 MeV proton cyclotron from the NIRS-Cyclotron-Facility. The study included the radioisotope production, gas separation, gas compression, gas pulsing, ionization in an ECR ion source and injection into the HIMAC synchrotron. It was estimated that with the developments discussed in that work, a ^11^C^6+^ beam with 1 x 10^8^ ppp intensity could be extracted from the HIMAC (85). However, in subsequent publications (86–88) production *via* the ^14^N(p,α)^11^C reaction and N_2_ gas targets was discarded due to high N_2_ impurities (~1 x 10^22^ for a 0.1 l target) overloading the ion source. Most recently, NIRS investigates ^11^C beam production *via* the ISOL method using solid boron-based targets (86, 88). Figure 1 shows the proposed ISOL-type ^11^C beam production system, comprising a solid NaBH_4_ target, a driver cyclotron providing 20 MeV protons, a molecule production and separation system (CMPS), a 1+ ion source, a mass separator and an Electron String Ion Source (ESIS) ion source for charge breeding. Since the HIMAC has an acceleration efficiency of 10% (from Linac injection to the treatment room) (86), and ~1 x 10^9^–1 x 10^10^ ^11^C ions are required for treatment, the ISOL system must be able to provide ~1 x 10^10^ ions extracted from the ion source system. Three boron-based targets have been tested (86, 89), using 18 MeV protons, a beam intensity of 18 μA for 20 min and an isotope extraction in form of ^11^CH_4_. Elemental boron showed highest in-target production yield, however, only 0.2% could be trapped as ^11^CH_4_. From a B_2_O_3_ target more than 76% of the initially produced ^11^C activity could be collected as ^11^CO_2_, but they report that the carbon oxide separation is too difficult (89). Finally, it was claimed that a NaBH_4_ target suited best their requirements with 5 x 10^12^ collected ^11^CH_4_ molecules, which corresponds to more than 29% of the in-target production yield. It was projected that this yield can be increased to the order of 1 x 10^13^ by increasing the proton beam intensity to 30 μA. One major concern of this approach is the low melting point of 400°C and the fact that the target thickness is chosen to absorb the entire proton beam. Consequently, serious complications with respect to heat damage and target endurance should be taken into account.

However, based on these findings, the CMPS was developed, comprising two cryogenic traps, which separate the molecular species according to their difference in vapor pressure. Depending on the impurity concentration, a collection/extraction efficiency of 60–80% is reported (92). As a result, the ion source system depicted in Figure 1 has to reach a total efficiency of 0.1% for the proposed 1+ to n+ ionization scheme. Currently, a singly charged ion source based on electron impact ionization is under development (84). Considering an average C^4+^ ionization efficiency of approximately 10% that was observed in an ESIS (93, 94), the 1+ ion source is designed to provide the required 1% ionization efficiency. However, it must be mentioned that the referred charge breeding efficiency was determined using stable, neutral CH_4_ gas which was frozen in a cryogenic cell. By heating the cell, the methane was evaporated and part of it was injected into the ion source. A recent study, performed at CERN, yielded that the Electron Beam Ion Source (EBIS) charge breeding efficiency using ion beam injection is considerably lower (95). Since an ESIS ion source is basically a modified EBIS, it is consequently questionable whether this ion source system (Figure 1) will accomplish the desired 0.1% overall ionization efficiency for high intensities.

## Required Accelerator Layout

All currently existing carbon therapy accelerators are of synchrotron type and are based on the PIMMS design (3). Their beam specifications at the irradiation room are summarized below:

- Ion species: C^6+^
- Beam energy: 120 to 400 MeV/u
- Beam intensity: ≤ 4 x 10^8^ particles/spill
- Spill duration: 0.1 to 10 s
- Repetition rate: ≤ 0.2 Hz.

To allow similar treatment times to the existing facilities, a ^11^C facility would need to deliver a comparable beam intensity per time unit. The main challenges to solve for a ^11^C facility are to reach the required ^11^C intensity and to assure a stable and reproducible performance. Several options are possible:

- Production of ^11^C *via* projectile fragmentation, from a beam at the final required energy
- Upgrade the existing design of a synchrotron-based carbon therapy accelerator, by supplementing the standard ^12^C injector by a ^11^C injector, able to inject the required intensities of ^11^C, with the time structure required by the existing synchrotrons.
- Accommodate the ^11^C injector to a Linac-based or cyclotron-based accelerator.

The review of the past results on the production of post-accelerated ^11^C ion beams (section Production of Carbon-11 Beams: Overview of Past Results of the present report) is showing that the production *via* the projectile fragmentation method (option A) cannot be considered for therapy, due to low production cross section and undesirable beam characteristics such as large momentum spread, large emittance and poor beam purity. The option C would allow a relaxation of the intensity constraints for the ^11^C injector, but it represents a “green-field” approach, as currently there is no such Carbon therapy accelerator in operation, due the challenges raised by the acceleration stage. We therefore focus in the present study mainly on the option B, with the goal of identifying and discussing the possible scenarios for the ^11^C injector. If a satisfactory solution can be implemented for the option B, it can in principle be easily adapted also option C.

**Figure 2:**
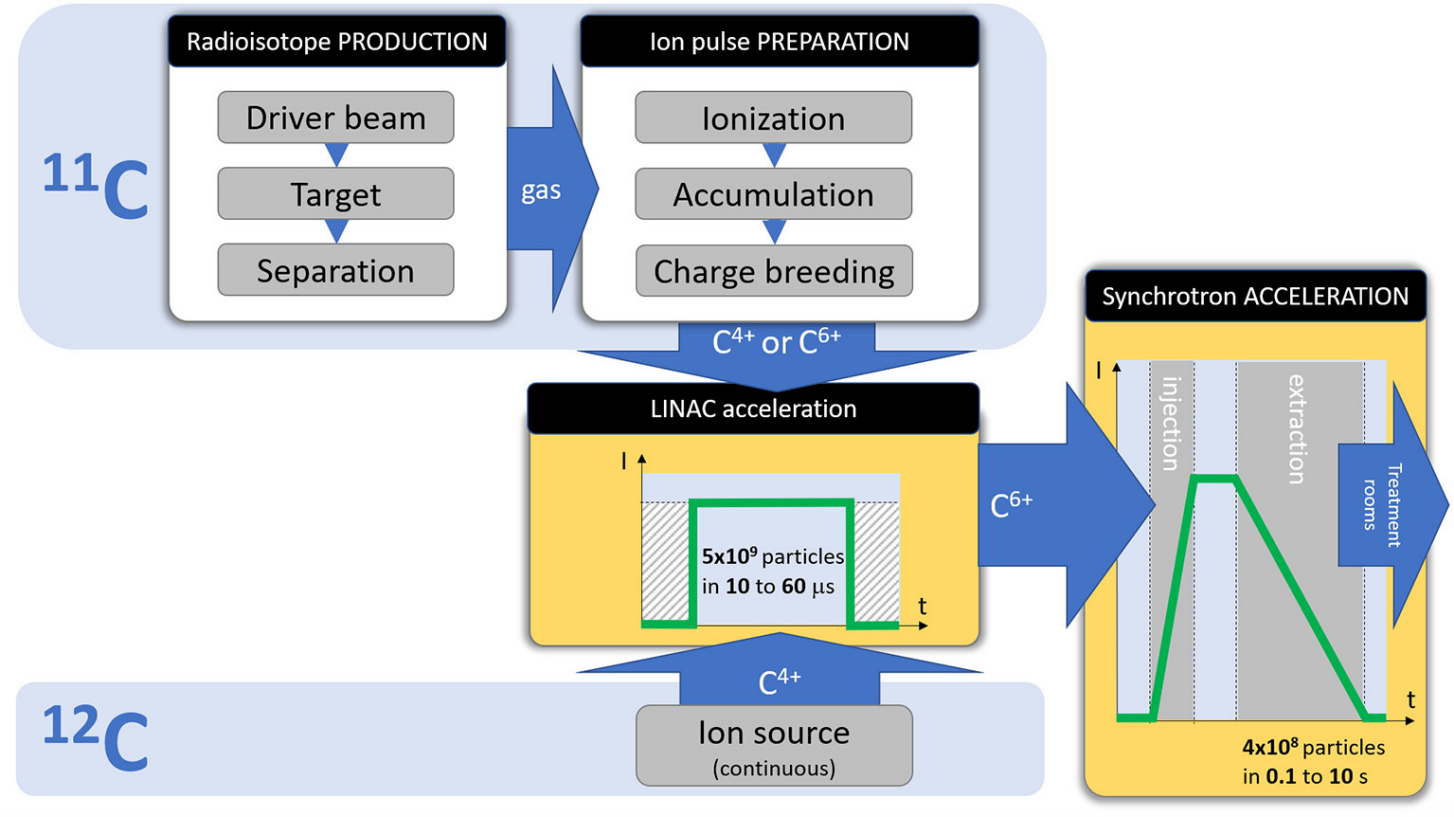
Required stages and beam parameters for a therapy accelerator, and the differences between a ^12^C and ^11^C injector.

Figure 2 shows the differences between a ^11^C and ^12^C injector, and how both must comply to the same pulse requirements for injection into the synchrotron. The focus of the present study is to analyse the feasibility of the ^11^C injector, with the steps presented in the dedicated ^11^C box of Figure 2:

- Radioisotope production (^11^C), achieved by irradiating a target with a driver beam, followed by isotope separation/purification. These production steps are analyzed in section Radioisotope Production of the present study.
- Preparation of the ion pulse for acceleration, consisting in ionization, accumulation (if needed) and charge breeding. These preparation steps are analyzed in section Ion Pulse Preparation of the present study.

The acceleration stages (Linac and synchrotron) are not detailed in the present study, as they do not present any specificity for the ^11^C case.

## Analysis by Accelerator Component/Stage

### Radioisotope Production

To produce ^11^C, a chosen target is irradiated by a primary light particle beam (driver), followed by isotope extraction from the target and purification (if needed) before the generation of the ion beam pulse to be sent to the Linac for acceleration.

Several possibilities are evaluated for the driver beam, the target and separation, considering their feasibility to achieve the required beam intensity and to be implemented into existing facilities.

#### The Driver Beam

Three options have been considered as relevant for the present study:

- 7 MeV protons with intensities up to 7 μA, which can be extracted from the Linac of existing therapy facilities.
- 18 MeV protons, as can be provided by a compact cyclotron used for industrial production of radioisotopes. There are several products commercially available. For instance, IBA's 18 MeV proton cyclotron CYCLONE®KIUBE, available in several editions that differ in beam intensity. Furthermore, IBA offers commercial N_2_ gas target solutions, as well as solid target stations (96);
- 250 MeV protons, as could be provided by a compact proton therapy cyclotron. Such a cyclotron could be used, on the one hand, to produce ^11^C for PET-aided hadron therapy. On the other hand, it may be used for conventional proton therapy. Consequently, such an approach would increase the throughput of the treatment facility. Such cyclotrons are commercially available, for instance the VARIAN ProBeam^TM^ 250 MeV, 0.8 μA superconducting cyclotron.

#### The Production Target

The choice of the target is an essential criterion for the accelerator chain. In principle, many choices of beam-energy-target combinations are possible, however mostly two types of beam-target combinations are conceivable with respect to the production of high intensity post-accelerated ^11^C beams: nitrogen gas targets and boron nitride targets.

**Figure 3:**
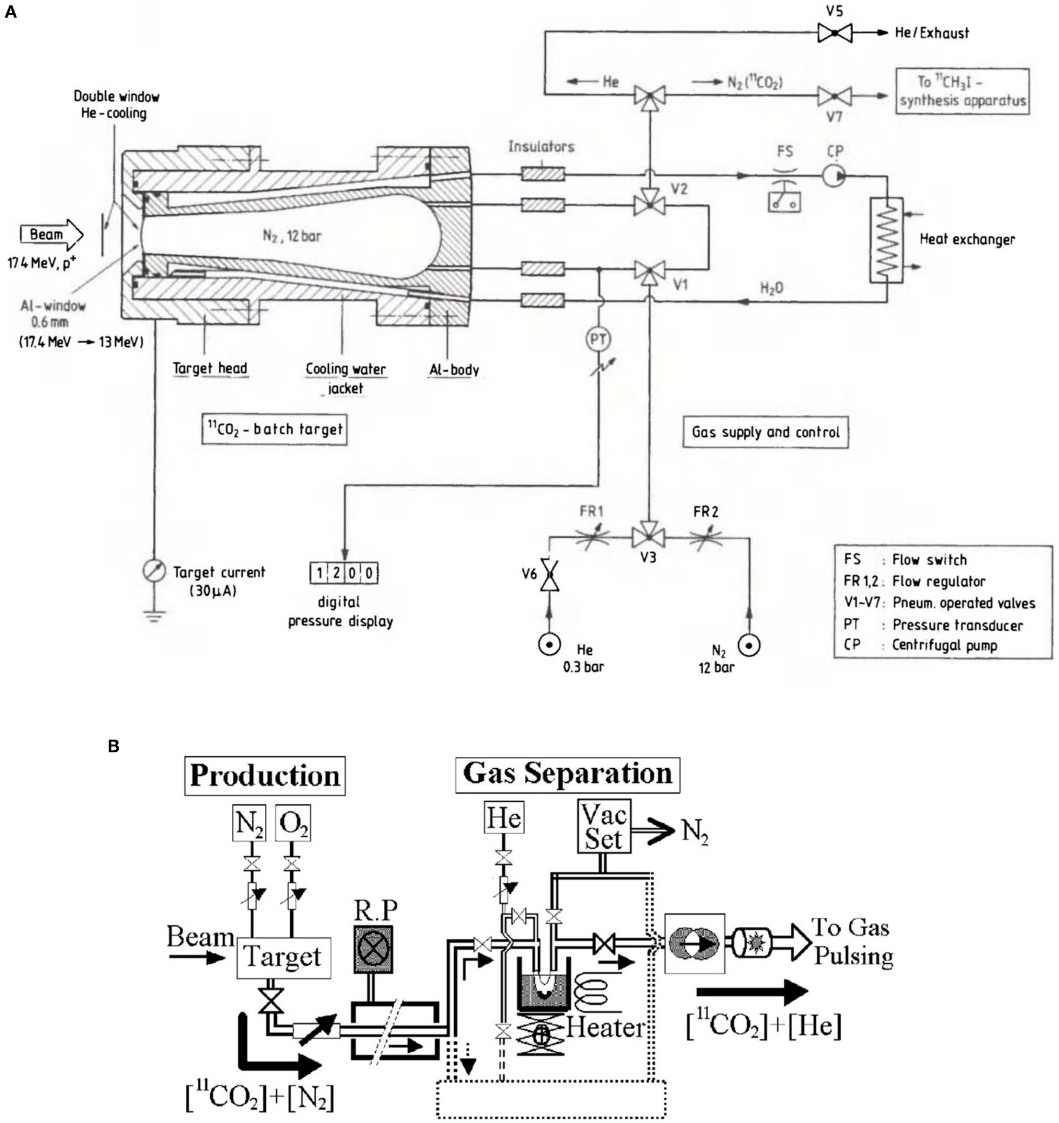
Examples for a typical N_2_ gas target system **(A)** and for a chromatography gas separation system **(B)** using a cryogenic trap (85, 90).

High-pressure N_2_ targets (several bar) are commonly used to produce ^11^C for PET-imaging. The gas cells are irradiated with a low-energy proton beam for a duration of several minutes. Subsequently, ^11^C is separated from the gas mixture by purging it through a chromatography gas separation (CGS) system. Usually, the ^11^C is extracted in molecular form, as CO_2_ or CH_4_. Two examples of typical gas target systems can be seen in Figure 3 (85, 90). It consists of a water-cooled conical cylinder holding the target gas and a tube system for the gas transport (Figure 3A). The target chamber is filled with high purity N_2_ gas to pressures usually greater than 10 bar. Oxygen or hydrogen is mixed in percentage amounts into the target gas to produce ^11^CO_2_ or ^11^CH_4_, respectively. Typical gas separation systems (Figure 3B) use cryogenic traps, i.e., stainless-steel tube/coil immersed in liquid nitrogen, which traps CO_2_. Increasing the trap temperature will then result in their release. Alternatively, chromatography columns (CH_4_) or molecular sieve (CO_2_) traps may be used. Chromatography columns, such as Porapak Q columns, separate CH_4_ from other species based on differential adsorption times on the column's material, which result in different flow rates (97). Molecular sieve traps are pre-activated columns of a selected microporous material, usually measured in Angstrom. Advantage of such traps is that they can trap CO_2_ at room temperature and release the captured molecule by heating the material to 100–200°C (97, 98). A general disadvantage of gaseous N_2_ targets is that ^11^C production is carried out in batch mode. As a result, to ensure a continuous operation, an automatized target loading/unloading system has to be established. To give the reader an idea, such a system was used in the BEARS project in Berkeley (2).

**Figure 4:**
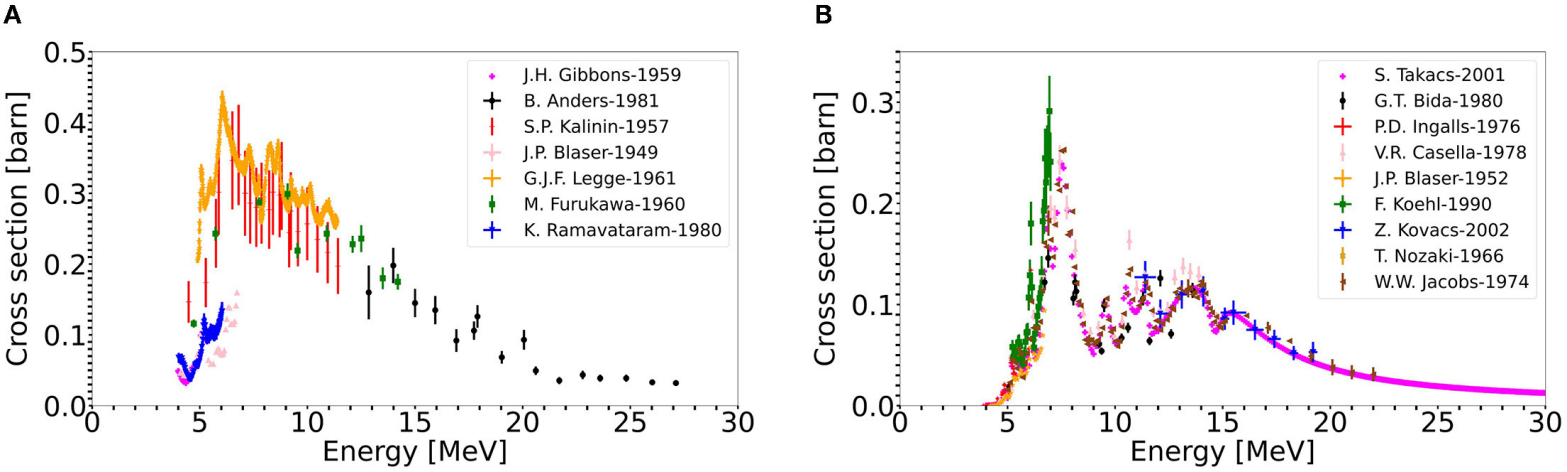
^11^C production cross sections from the proton induced reactions **(A)** ^11^B(p,n)^11^C, **(B)** ^14^N(p,α)^11^C (91).

The second possible approach to produce large quantities of ^11^C is using a solid, boron comprising target. Boron, in comparison with nitrogen, has a higher cross section to produce ^11^C. The corresponding cross sections, ^11^B(p,n)^11^C and ^14^N(p,α)^11^C are presented in Figure 4 (91). By using a solid target, higher in-target production yields can be expected, because of the higher cross section and given the material's higher density. Furthermore, a solid target eases target handling and radioactive waste management. As introduced earlier, ISOLDE produces since more than 50 years radioactive ion beams *via* the ISOL method. In this technique, usually, solid targets are irradiated with a proton driver. The isotope of interest is produced, among others, inside the material. By heating the target close to its melting point, the isotopes are evaporated from the material's surface. High temperatures are an essential criterion, as isotope diffusion and effusion processes depend exponentially on temperature. Once the isotopes are released from the target material, they are separated as will be described in the next section.

In the prospect of producing high intensity mass separated ^11^C beams, a boron nitride (BN) ISOL-type target was developed and characterized (99). The target was manufactured to provide a controlled microstructure, for short diffusion and effusion times, to enhance the isotope release properties. The isotope release is often considered as a bottleneck in intense RIB production (100, 101). Furthermore, it is foreseen to operate this target with a controlled oxygen leak to extract ^11^C in the form of CO. Molecular isotope extraction further increases the release efficiency, as carbon is very refractory and easily forms strong bonds with hot metal surfaces.

The expected in-target production yields for the different target-driver combinations can be calculated. For this purpose, simulations were performed with the particle physics Monte Carlo simulation code FLUKA (48, 49).

The target geometries used as an input for the simulations are the following:

- In the case of the N_2_ gas target, a geometry based on the commercial IBA target (102) was used. This comprises a 40 cm^3^, conical target container filled with a 20 bar N_2_/O_2_ (0.99/0.01 vol%) gas mixture. For the simulation, a 600 μm thick aluminum entrance window was assumed, which is important to address as the proton beam will lose energy in this window.
- In the case of the solid BN target, a cylindrical target pellet was used, with a diameter of 30 mm. The target thickness is varied in each case, such that the proton beam exits the target pellet with a remaining energy of 4-5 MeV. With such a configuration, the deposited power can be reduced, while the in-target production yield is only marginally reduced. This effect is shown in Figure 4, where it's visible that both cross sections significantly drop for energies lower than 5 MeV.

Table 2 gives a comparison of the expected ^11^C production yields for the different driver-target scenarios. The produced in-target yield is given as an End-Of-Beam (EOB) yield for the N_2_ target, as this target is operated in batch mode. For the BN target, operated on-line (in cw mode), the quoted yield is the saturation yield reached after 1.5 h.

**Table 2:** Summary of the selected properties (driver beam and of the production target) and comparison of in-target production yields for different driver-target combinations.

	**7 MeV Linac**	**CYCLONE^®^ KIUBE**	**VARIAN ProBeam™**
**Properties of the driver beam**			
Particle	proton		
Energy [MeV]	7	18	250
Intensity [μA]	7	50	0.8
Pulse structure	pulsed	dc/pulsed	dc/pulsed
Beam cross section [cm^2^]	Gaussian, 8.24		
Batch/extraction time	BN: cw		
[min]/cw	N_2_: 30 min irradiation + 4 min trapping + 1 min release		
**Properties of the production target**			
Batch/extraction time	BN: cw		
[min]/cw	N_2_: 30 min irradiation + 4 min trapping + 1 min release		
Target size, geometry	BN: cyl. 7.1 x 0.2	BN: cyl. 7.1 x 0.29	BN: cyl. 7.1 x 34.2
S [cm^2^], L [cm]	N_2_: conical, 40 cm^3^		
	1.8 x 11.3 x 5.7		
Density [g/cm^3^]	BN: 1.3		
	N_2_: 0.02332		
Deposited beam power	BN: 49	BN: 700	BN: 148
[W]	N_2_: 3	N_2_: 620	N_2_: 0.7
Target temperature [°C]	BN: 1500		
	N_2_: n/a		
Saturation yield, Y_sat_	BN: 1.7	BN: 11.9	BN: 79
[GBq/μA]	N_2_: 0.2	N_2_: 8.2 (5.5)	N_2_: 0.7
In-target yield	BN: 12	BN: 593	BN: 63
EOB/Saturation [GBq]	N_2_: 0.7	N_2_: 262 (176)	N_2_: 0.35
Release efficiency [%]	BN: 10		
	N_2_: 80		
Molecular sideband	BN: CO		
	N_2_: CO_2_		
Impurities	BN: N_2_, Ar, O_2_		
	N_2:_ N_2_, O_2_, NO_x_		

The release efficiency of the BN target was experimentally determined (103), whereas the efficiency for the N_2_ target was calculated. The listed impurities are only the ones originating from the target material itself and its operation. In the BN case, Ar refers to the carrier gas used with the controlled O_2_ leak.

Table 2 shows that the N_2_ gas target generally offers lower yields compared to the BN target, which mainly can be attributed to the higher cross sections when exploiting the (p,n) reaction channel on boron. The discrepancy between these two targets is more pronounced for the 7 MeV Linac and 250 MeV cyclotron as the commercial gas targets are not designed for these energies and consequently do not utilize the nitrogen ^11^C production cross section adequately. In detail, the 7 MeV Linac loses up to six MeV within the aluminum entrance window, which implies that it is arguable whether any ^11^C is produced at all, as the cut-off energy of the ^14^N(p, α)^11^C reaction is around 3 MeV. The FLUKA simulated yields of the LEBT-Linac-N_2_ gas target combination should be therefore treated with caution. The 250 MeV cyclotron on the other hand, deposits merely 1 MeV within the N_2_ target gas, which explains the low yields. Both 7 MeV Linac and 250 MeV cyclotron would benefit from an optimization of the N_2_ gas target design.

From Table 2, we can see that the 250 MeV VARIAN ProBeam–BN target combination offers the highest saturation yield, when normalized to the primary proton beam current, since a stack of many target pellets is required to slow down the proton beam below 5 MeV (34 cm). However, the CYCLONE®KIUBE cyclotron driver option presents the highest achievable in-target yield due to the much higher proton beam intensity. In standard edition, beam currents up to 150 μA are possible (102). The produced yield scales linearly with the proton current, but the selection of the primary proton current I should be handled with caution as low-energy proton beams deposit considerable power into the target, which result in rapid heating of the target material. Generally, the ISOL-type targets are operated at high temperatures (close to their melting point), to enhance diffusion and effusion processes. Previous studies (99) investigated the high-temperature stability of the BN target in typical ISOL operational conditions. In this respect, BN dissociation was expected at temperatures above 1,000°C. High-temperature studies, probing the developed BN target at temperatures up to 1,500°C demonstrated its applicability in such conditions (99). Eventually, the maximum applicable beam current for the BN target will depend on how efficient the target can be cooled. The commercial IBA N_2_ target is in practice operated with a 50 μA beam current. In the case of BN, such a beam current would correspond to a power deposition of 700 W. We assume at this point that target cooling techniques are able to prevent the target to exceed the 1,500°C maximum temperature.

#### The Isotope Separation

Separation of ^11^C from impurities depends on the target-driver combination, the molecular side band in which ^11^C is produced, the impurity species and their quantities. The appropriate separation modality is strongly influenced by the requirement of the next stage (ion pulse preparation) of having the amount of ^11^C not surpassed by orders of magnitude from impurities. For this, there are three possibilities:

- Direct coupling to next stage (ion pulse preparation), i.e., no separation is required
- Application of a CGS system to isolate CO_2_ from other species
- ISOL-type separation, using a 1+ ECR ion source, electromagnetic mass separation and a deaccelerator.

Direct target coupling would of course be the favorable route, as it would not require further components. If the amount of impurities is considerably larger, chemical separation (e.g., CO_2_ from N_2_) by means of a cryogenic or molecular sieve trap may be applicable. On the other hand, if a separation from same chemical elements is required, an electromagnetic mass separation (which enables isotopic separation due to the A/q selectivity) needs to be used.

The nitrogen gas targets are filled to 20 bar, which will further increase during irradiation, therefore a direct coupling is not possible. Consequently, some sort of trapping needs to be implemented, to separate the macroscopic quantities of N_2_ and O_2_ from the active target gas. It is therefore reasonable to use a CGS system to separate ^11^CO_2_ from these impurities (as already presented in the typical layouts, Figure 3). Since ^11^C is produced in the CO_2_ molecular sideband, a cold trap or molecular sieve trap may be used, whereas the commercially available N_2_ gas target is usually equipped with the former type (102). The trapping efficiency of cold traps depends on the surface area of the coil that is immersed in liquid nitrogen. Usually trapping efficiencies >95% are achievable (104). The trapping of CO_2_ is carried out over a duration of ~4 min, followed by 1 min of heating to release the trapped molecules. Hence, one batch of ^11^C is produced and purified in 30 + 4 + 1 min. By considering the decay during trapping, one obtains an isotope specific separation efficiency. Considering the decay of ^11^C with T_1/2_ = 20.4 min, approximately 80% of the produced ^11^C can be recovered as ^11^CO_2_. Assuming a transport efficiency to the next stage (ion pulse preparation) of 50%, an overall efficiency of 0.4 can be achieved for ^11^C. One drawback of such a cold trap is that N_2_, O_2_, NO_x_ and CO are trapped to some extent as well (98, 104). Using liquid argon instead of liquid nitrogen resolves the trapping of N_2_ (104). Alternatively, when using a molecular carbon sieve trap, equal efficiencies can be achieved, while O_2_, CO, or NO are probably not retained (98). Consequently, a molecular sieve trap might be better suited, since it is not clear how much N_2_, O_2_ and NO_x_ is trapped together with the ^11^CO_2_ in the CGS system. As mentioned earlier, if these species exceed the quantity of ^11^CO_2_ by an order of magnitude or more, this will be problematic for stage of ion pulse preparation.

**Figure 5:**
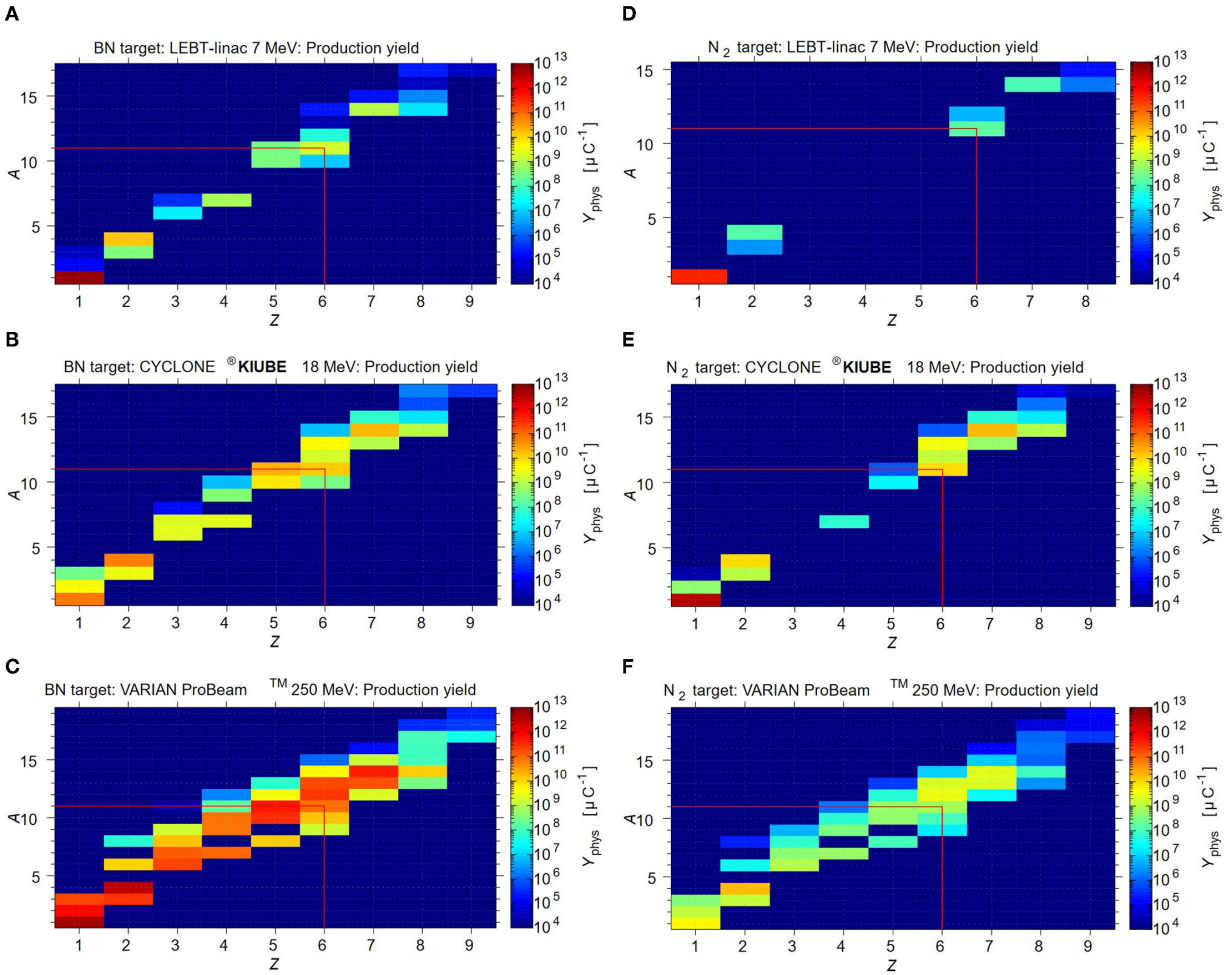
FLUKA simulations for the physical production yield for the different target+beam options. The yield is expressed in nuclei per μC. **(A)** BN target +7 MeV beam, **(B)** BN target + 18 MeV beam, **(C)** BN target + 250 MeV beam **(D)** N_2_ target + 7MeV beam, **(E)** N_2_ target + 18 MeV beam, **(F)** N_2_ target + 250 MeV beam.

Besides the contamination resulting from the target material itself (N_2_ and O_2_), other (radioisotopes are produced during irradiation, which must be considered. Figure 5 shows the simulated physical thick in-target production yields, expressed in nuclei per μC, for an IBA-type N_2_ target in combination with the different driver options. Minor contaminations are expected for 7 MeV LEBT-Linac due to the low driver beam energy. When employing the more energetic 18 MeV or 250 MeV cyclotrons, considerably more elemental by-products are generated during irradiation, which may form molecules such as NO_2_F, FNO and H_2_O. These molecules should easily be removed from ^11^CO_2_ using a cold or molecular sieve trap. However, among ^11^C, other carbon isotopes are produced as well, which cannot be separated by a CGS system due to their identical chemical properties. Depending on their quantity, further separation may be required before the next accelerator stage. Table 3 provides an overview of the expected gas output per batch of a N_2_ target after CGS-type separation, depending on the proton driver.

**Table 3:** Gas output (molecules per batch) after separation stage for an N_2_ target: ^11^C molecular compounds (in bold) and the corresponding impurities.

	**7 MeV**	**CYCLONE^®^**	**VARIAN**
	**Linac**	**KIUBE**	**ProBeam™**
**Properties of the isotope separation**			
Separation technique	CGS		
Separation efficiency [%]	40		
Molecular sideband	CO_2_		
**Output of separation stage (molecules)**			
^10^CO_2_	-	-	2 x 10^4^
^**11**^ **CO**_**2**_	**5 x 10** ^**11**^	**2 x 10** ^**14**^	**2 x 10** ^**11**^
^12^CO_2_	3 x 10^10^	5 x 10^13^	2 x 10^12^
^13^CO_2_	-	1 x 10^14^	1 x 10^12^
^14^CO_2_	-	3 x 10^10^	7 x 10^9^
N_2_, O_2_, NO_2_	Unknown	Unknown	Unknown

When using the 7 MeV LEBT-Linac or the 18 MeV CYCLONE®KIUBE cyclotron, the number of produced carbon impurities is either lower or of the same order of magnitude. A direct coupling is therefore feasible, provided that potential trapping of target gas residuals (N_2_, O_2_ and NO_x_) is limited. However, when using the 250 MeV VARIAN ProBeam^TM^ cyclotron for isotope production, Table 3 suggests that ^12, 13^C are exceeding ^11^C production by one order of magnitude, which is perceived as inadmissible if not a further separation modality is applied.

In the case of the BN target, its operation will result in a continuous gas flow. To evaluate whether a direct coupling to the next stage is feasible, one needs to estimate the ^11^CO yield, as well as the amount of impurities that are comprised in this gas flow. The impurities for this target originate from three sources:

- The radioisotopes generated during target irradiation which are evaporated from the target together with the ^11^CO
- The vapor pressure of the target material due to the 1,500°C target temperature
- The applied gas leak of 1 x 10^−5^ mbar l/s using a gas mixture of Ar/O_2_ (90/10 vol.%). The gas leak is applied to enhance the isotope release from the target matrix, with the O_2_ serving to create an oxidizing atmosphere, while the Ar is added due to safety regulations.

We first discuss the expected ^11^CO yield and the (radio-) isotope impurities. Figure 5 shows the FLUKA simulated physical thick in-target production yields, expressed in nuclei per μC, considering the BN target employed with the different production driver. The release efficiency for ^11^CO is calculated to be of 10% and the saturation is reached after 1.5 h. The saturation of stable carbon isotopes will require significantly more time, as no decay is occurring. Therefore, to be able to compare, the expected yields of stable and long-lived carbon radioisotopes are calculated assuming *t* = 16 h of irradiation. This duration is chosen, considering that in practice the synchrotron is started during the night for beam commissioning and accounting for a full day of operation for therapy. The reader is referred for more information to the corresponding release study (103). The summary of the expected gas output of the BN target due to evaporated radioisotopes is shown in the top part of Table 4.

**Table 4:** Gas output (molecules *per second*) from the BN target before separation: ^11^C molecular compounds (in bold) and the corresponding impurities.

	**7MeV Linac**	**CYCLONE^®^ KIUBE**	**VARIAN ProBeam™**
**Carbon isotopes**			
^9^CO_2_	-	-	7 x 10^5^
^10^CO_2_	6 x 10^5^	1 x 10^8^	1 x 10^8^
^**11**^ **CO**_**2**_	**1 x 10** ^**9**^	**6 x 10** ^**10**^	**6 x 10** ^**9**^
^12^CO_2_	2 x 10^8^	5 x 10^10^	1 x 10^11^
^13^CO_2_	1 x 10^5^	1 x 10^11^	7 x 10^10^
^14^CO_2_	8 x 10^5^	2 x 10^8^	2 x 10^9^
^15^CO_2_	-	-	2 x 10^3^
**Target evaporation**			
N_2_	2 x 10^14^	2 x 10^14^	2 x 10^14^
**Support gas**			
O_2_	2 x 10^13^	2 x 10^13^	2 x 10^13^
Ar	2 x 10^14^	2 x 10^14^	2 x 10^14^
He	9 x 10^10^	3 x 10^12^	2 x 10^12^

In conclusion, the 7 MeV LEBT-Linac produces ^*x*^CO impurities of comparable magnitude, whereas the 18 MeV CYCLONE®KIUBE and the 250 MeV VARIAN ProBeamTM cyclotron, generate isotopic contaminants that exceed ^11^CO by one or two orders of magnitude. In the former case, it has to be investigated whether such a load is admissible for direct injection to the next stage, whereas the latter case most likely prevents the direct injection. Besides carbon, volatile isotopes are produced inside the target (see Figure 5) that are expected to be released very efficiently. Isotopic nitrogen and oxygen will only be traces compared to the impurities resulting from target operation, which will be discussed henceforth. However, hydrogen and helium isotopes are produced in significant amounts, which will most likely be a limiting factor for the next stage. The release of hydrogen is difficult to evaluate as it may form chemical compounds such as HBO. Helium on the other hand should be simply released, where the bottom part of Table 4 indicates the maximum extent by assuming a 100% release efficiency.

The second source of impurities stems from target operation at *T* = 1,500°C and is attributed to the vaporization of N_2_ due to BN dissociation: 2BN → 2B + N_2_(g). This feature was investigated in dedicated high temperature stability studies at the ISOLDE off-line laboratories (99), which showed that no N_2_ evaporation is detectable at a base pressure of 5 x 10^−7^ mbar. Assuming ideal gas conditions, room temperature in the gas transfer line and using the 300 l/s throughput of the employed turbo vacuum pump, <3 x 10^15^ particles per second are vaporized from the target. A residual gas analysis showed that N_2_ accounts for approximately 7% of the total residual gas composition, which corresponds to 2 x 10^14^ N_2_ molecules per second. This is significantly exceeding the expected ^11^CO yield for all possible proton driver, therefore eliminating a direct coupling scenario.

The third source of impurities originates from the application of the 1e^−5^ mbar l/s calibrated gas leak using an Ar/O_2_ (90/10 vol.%) gas mixture. Such a controlled gas supply contributes twofold to the impurities: firstly, a net gas flow of 2 x 10^14^ molecules per second is associated to the corresponding leak, assuming ideal gas conditions and room temperature in the gas transfer line. It is worth emphasizing that an Ar/O_2_ gas mixture is employed due to safety regulations, preventing injection of pure O_2_, which could reduce the gas leak by one order of magnitude while maintaining the same oxygen potential. Moreover, argon could be replaced by helium, considering the charge space limitation of the EBIS charge breeder. Secondly, BN is sensitive to oxidation at high temperatures, resulting in further N_2_ evaporation: 2BN + 3/2O_2_(g) = B_2_O_3_(l) + N_2_(g). A quantitative analysis of the oxidation kinetics of such target operation indicated that the external O_2_ supply results in < 12% enhanced N_2_ evaporation, which is insignificant considering the order of magnitude estimation that is discussed at this stage.

In summary, Table 4 shows the expected continuous gas output that originates from the BN target, including all types of impurities. As the impurities are exceeding the ^11^CO flow significantly for all of the discussed proton driver, it is necessary to incorporate a separation modality prior to next stage. The separation technique may be either CGS-type or ISOL-type.

The ^11^CO separation using a CGS system is challenging as the main impurities are N_2_, O_2_ and the carrier gas (Ar) of the oxygen leak. CO and N_2_ have very similar molecular properties which complicate the separation process. The typical cold traps and molecular sieve traps described earlier are not suited for efficient CO trapping, since CO is, at most, only partly captured (98). There exist a variety of other systems or materials (105) that are used to purify CO-containing gas mixtures. However, often they work under high pressures or they trap significant amounts of N_2_ and O_2_ as well, while CO is only trapped to 23%. Alternatively, one could oxidize ^11^CO to ^11^CO_2_ for which the CGS systems described earlier can be applied. Studies on high-temperature CO oxidation suggest that high conversion efficiencies can be achieved in short times (106). In the aforementioned study, hot inert (N_2_) carrier gas was sent through a cylindrical quartz duct in which CO and water were rapidly mixed. Best results were obtained at temperatures 1,100°C with a water mole fraction of 0.0248. Conversion efficiencies close to 100% were found in a time span <1 s. If we consider such an approach for CO oxidation, this prevents the subsequent application of a cold trap for CO_2_ separation as water will condense as well. However, carbon molecular sieve traps have a low affinity for water and therefore are a suitable option (98). One drawback of such a separation route is again the fact that trapping results in a batched injection.

To calculate the amount of recovered isotopes when employing the BN target with such a modified CGS system, one has to account for the accumulation and simultaneous decay of radioisotopes. Considering the application of a molecular sieve trap instead of a cold trap, due to their similar working principle, we can apply the same trapping mechanism as discussed for the cold traps, i.e., trapping and release time of 4 and 1 min, respectively with a trapping efficiency larger than 95%. An overall efficiency of approximately 6% is calculated for ^11^CO_2_. Stable carbon contaminants resulting from the BN target will not decay during the 4 min of accumulation and will subsequently be transported to the next stage with an efficiency of 50%. As a result, a deterioration of the ^11^CO_2_ to carbon impurity ratio will occur. This feature should be addressed when considering the possible application of a CGS system combined with the BN target, as the impurity level should not exceed the amount of ^11^CO_2_ molecules significantly. The expected load of the BN target-CGS system combination is shown in Table 5.

**Table 5:** Expected ^11^C available after the separation stage for a BN target, together with the corresponding impurities.

	**7 MeV Linac**	**CYCLONE^®^ KIUBE**	**VARIAN ProBeam™**
**Properties of the isotope separation**			
Separation technique	CGS		
	ISOL		
Separation efficiency (%)	CGS: 6		
	ISOL: 5		
Molecular sideband	CGS: CO_2_		
	ISOL: CO		
^11^ **C at output of separation stage (molecules)**			
CGS	1 x 10^11^/batch	6 x 10^12^/batch	7 x 10^11^/batch
ISOL	6 x 10^7^/s	3 x 10^9^/s	3 x 10^8^/s
**Residual impurities**			
CGS: ^X^ CO_2_	2 x 10^10^	~ 2 x 10^13^	~ 2 x 10^13^
ISOL: ^13^N^14^N	<2 x 10^3^	<2 x 10^9^	<6 x 10^9^

An ISOL-type electromagnetic mass separation system may be used to isolate ^11^CO from other impurities. This option can be interesting especially because the BN target was developed as an ISOL target. This method relies on the use of a suitable ion source for efficient 1+ ionization and of a dipole magnet with high resolving power. The reference values we use for the 1+ ionization efficiencies of CO and CO_2_ are respectively 14 and 4%, as reported in (76) for a 2.45 GHz ECR ion source (MONO 1000) developed for efficient 1+ ionization at GANIL and reproduced and tested in an off-line study at ISOLDE.

Dipole magnets, tailored for the A/q of interest typically have separation efficiencies of 90%. Possible residual beam impurities are ^13^N^14^N since it shares the same A/q ratio when ionized to the 1+ charge state.

### Ion Pulse Preparation

As introduced in Figure 2, the main steps needed to be performed for the preparation of the ion pulse are the ionization (typically 1+), accumulation (typically as ions, but depending on the encountered limitations, transformation in neutral particles might be necessary) and the charge breeding (to 4+ or 6+ charge state). A complete solution will need to address all these steps, but not necessarily in this order (as is also the case in the following analysis), due to the numerous technical constraints to be addressed.

This section summarizes the results presented in (95). The goal is to discuss the possibilities of using a charge breeding scheme, that is ionization of ^11^C to 6+ charge state, based on an Electron Beam Ion Source (EBIS) for the preparation of the ^11^C beam. Test measurements under extreme operating conditions were conducted at the REX-ISOLDE facility to explore the limitations of the charge breeder for high-intensity, low-repetition-rate, molecular CO^+^ beams. Based on these findings, different possible scenarios of coupling a charge breeder with a therapy accelerator are discussed.

#### Setup and Methodology

**Figure 6:**
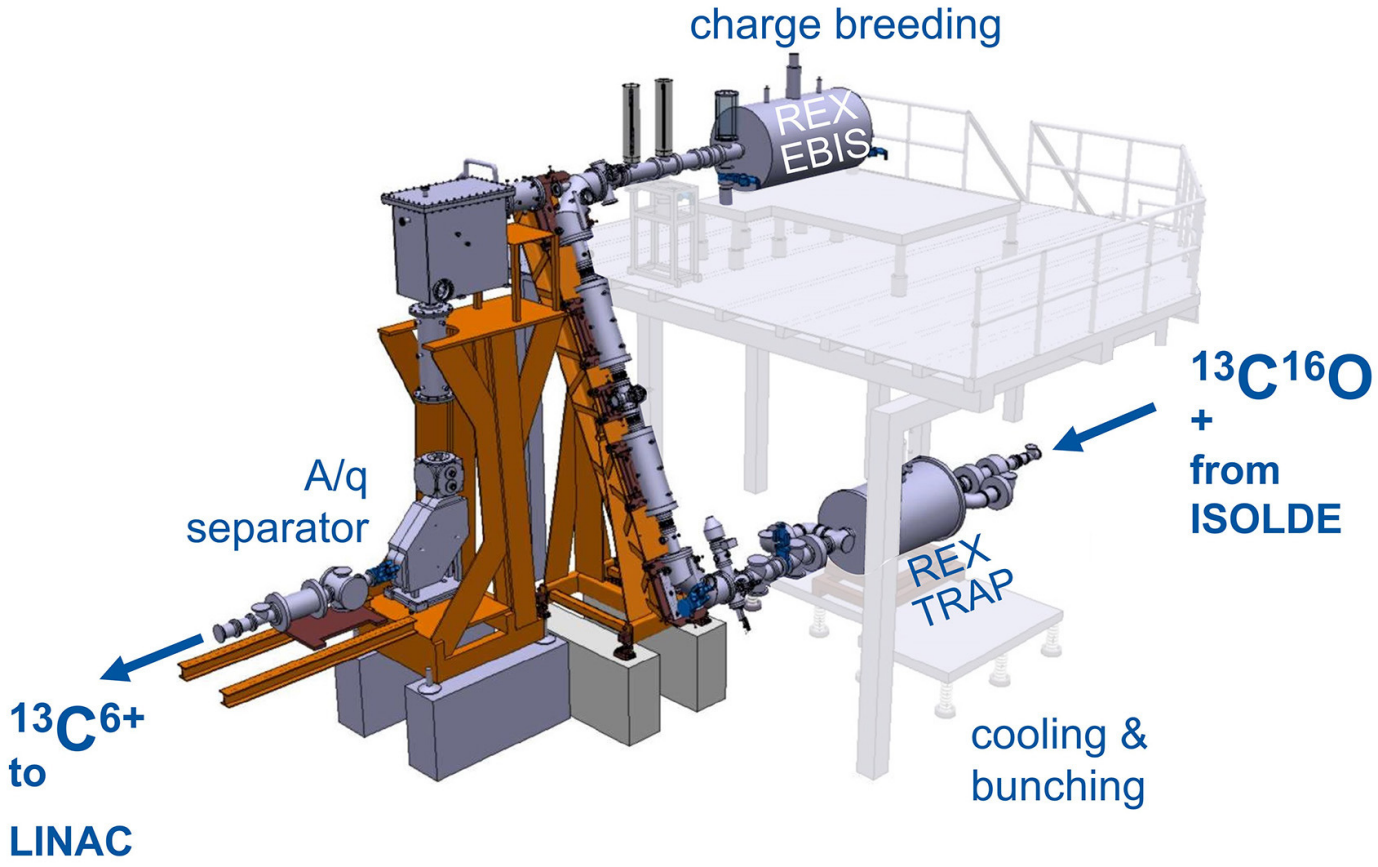
REX-ISOLDE low energy stage, comprised of REXTRAP for cooling and bunching of the 1+ ion beam, the electrostatic beam transfer section (BTS), REXEBIS for charge breeding 1+ → N+ and an A/Q separator. The charge breeder stage transforms a continuous 1+ beam into a pulsed beam of higher charge.

The concept of accumulation, breeding and post-acceleration of radioactive carbon beams was tested at REX-ISOLDE (107, 108), which is part of ISOLDE. Here, ISOL-produced radioactive beams are prepared in a charge-breeding stage (see Figure 6) before acceleration in the HIE-ISOLDE linac (4) and further transfer to the experimental stations. The charge breeding stage consists of two main devices, namely a Penning trap and an EBIS. The Penning trap, REXTRAP (109, 110), cools and bunches the quasi-continuous beam from ISOLDE. The bunched beam is transported *via* an electrostatic transfer section and injected into REXEBIS (111, 112), where the ions' charge state of initially 1+ is increased for an efficient post acceleration. After separation by A/Q in a Nier-type spectrometer (113), the selected beam is accelerated in the HIE-ISOLDE Linac.

At ISOLDE, the efficiency of the charge breeder stage is of major concern. Typically, rare isotopes with small production cross-sections are handled, hence, ion intensities are relatively low (ranging from a few ions/s to >1 x 10^8^ ions/s). In contrast to ISOLDE, a beam preparation stage for hadron therapy has to deal with considerably higher intensities. The efficiency will still play a central role in the design, as the production of the radioactive ions is limited. Furthermore, a synchrotron-based treatment facility would require long storage times of the 1+ ions, which is an additional challenge.

In the detailed report (95), different ways of using a charge breeding stage as a CO beam preparation tool for hadron therapy with a synchrotron have been laid out and investigated with regard to their feasibility and technical limitations. Measurement data was taken at ISOLDE to quantify the behavior and limitations of the Penning trap and EBIS under the extreme conditions of high-intensity, low repetition-rate beams and constraints due to molecular beams. The assumed requirements of the charge breeding stage are summarized in Table 6, and as seen the targeted output intensity is larger compared to the one introduced in Figure 2, as a margin to account for the fact that matching this type of injector to a synchrotron was not yet tested as a whole.

**Table 6:** Summary of the required functions of the charge breeding system for a hadron therapy facility based on a synchrotron, as used in (95).

**Functions of the charge breeder system**	**Details**
Accumulation of CO beam	During >1 s
Molecular breakup	CO → C + O
Charge breeding	C^4+^, C^5+^ or C^6+^
Extracted pulse length	<100 μs
Output intensity	1 x 10^10^ ions
Maximum emittance for C^4+^, 95% at 30 kV	~180 mm mrad

All measurements were performed with stable beams, either from the ISOLDE General Purpose Separator GPS (114), or from the local off-line surface ion source in front of the Penning trap (only K, not CO) (115). ^13^CO^+^ beams were produced in an ISOLDE target ion-source unit by injecting ^13^CO gas into a Versatile Arc Discharge Ion Source VADIS (116) *via* a leak. The measurements were performed with stable ^13^CO^+^ as radioactive ^11^CO^+^ beams with sufficient intensities cannot be reached with the present ISOL-system. It is assumed that the behavior of the radioactive ions is similar to that of the stable beam. For a radioactive beam, slightly higher loss rates in the Penning trap are expected due to the radioactive decay. However, as the half-life of ^11^C is relatively long (T_1/2_ = 20.4 min), only a small fraction of the ions decays during the storing time in the trap (decay constant 5.7 x 10^−4^ per second). Concerning the space charge limitation, the Brillouin limit for the Penning trap is inversely proportional to the ion mass, therefore the results can be scaled with the mass difference between ^13^C and ^11^C. In the EBIS no difference in capacity is expected between radioactive and stable beam as it depends only on the charge and not on the mass of the ions, to the first order.

Furthermore, the breakup of CO was studied for different trap configurations and buffer gases.

#### Pulsed Injection Into the EBIS

At the start of this study, pulsed injection into the EBIS with prior cooling and bunching in a Penning trap had been proposed as charge breeding scheme for a synchrotron-based ^11^C therapy facility (117). Within the investigations presented in (95), however, we have found that its working range is strongly limited, which makes it unsuitable for a therapy purpose.

Nevertheless, this scheme serves as an important reference case as it represents the normal operating scheme of the charge breeder system. We describe this operation case in the following of this section, together with the most important results from (95).

When injecting CO+ into REXTRAP, energy is transferred between the injected beam and the neutral buffer gas atoms through collisions. If the energy in the center-of-mass frame of the collision exceeds the dissociation energy of the molecule, there is a possibility that the molecule breaks up into carbon and oxygen. In principle, the CO molecule has to be broken up at some point in the charge breeding system. Therefore, it would be favorable if all molecules could be broken up in the trap such that all oxygen can be removed and an ion beam of atomic carbon is injected into the EBIS, thereby reducing the occupied space charge in the EBIS. The problem, however, is that when the CO+ dissociates in the trap, it is not guaranteed that the carbon atom remains positively charged. In the breakup there are two possible exit channels (95):

- CO^+^ → C^+^ + O (neutral)
- CO^+^ → C (neutral) + O^+^

where the branching ratio depends, among other things, on the collision energy with the neutral atom, with higher energies leading to an increased O^+^/C^+^ ratio (95). In the second channel, the carbon atom is neutralized and lost. When breakup happens, three beam components can exit the Penning trap: C^+^, O^+^ and CO^+^. The beam transfer section (BTS) from the Penning trap to the EBIS is completely electrostatic, so all beams can be transferred to the EBIS and the acceptance window in time is sufficiently large to accommodate the difference in flight time.

**Figure 7:**
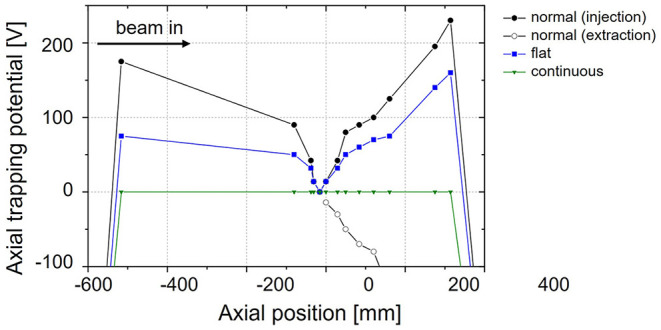
Different configurations of the axial trapping potential in REXTRAP. In the normal trapping configuration, the beam enters from the left side several 10 eV above the barrier or approximately 200 eV above the central trapping electrode (95).

In tests with molecular CO^+^ beams in REXTRAP, the influence of different parameters such as the injection energy, cooling time and choice of buffer gas on the trapping efficiency and breakup were investigated (95), with the following conclusions:

- For the buffer gas, two options were considered: He and Ne. Due to the significantly lower over-all efficiency observed, the idea of using He as buffer gas was discarded. All the further measurements were taken with Ne as buffer gas.
- The breakup of the CO^+^ molecules inside the Penning trap can partly be avoided by lowering the injection energy into the trapping region. In the normal trap configuration most of the molecules break up, hence, the beam is cooled on A = 13 as atomic carbon ions make up the largest part of the beam extracted from REXTRAP. In the flat trap configuration (see Figure 7), the injection energy is lower in order to reduce the breakup upon injection into the buffer gas, therefore the beam is cooled on A=29 and mostly CO^+^ molecules are extracted from REXTRAP.
- However, the normal trap configuration has a higher transmission than the flat trap, due to better injection conditions, and faster cooling during the first axial oscillation. For both flat and normal trap configurations, the trap transmission decreases with longer period times due to two effects. First, for longer holding times in REXTRAP the ions suffer more from the high loss rate discussed above. The CO^+^ beam is lost exponentially with a half-life of around 100 ms. The mechanism behind the losses has not been fully explained. Second, space charge effects in REXTRAP become more relevant, as the injection is continuous and higher integrated intensities need to be accumulated during longer period times (e.g., 2.8 x 10^8^ charges are injected for a 500 ms period time). When the accumulated charge per pulse approaches, and exceeds, the space charge limit of the Penning trap, the efficiency decreases.

**Figure 8:**
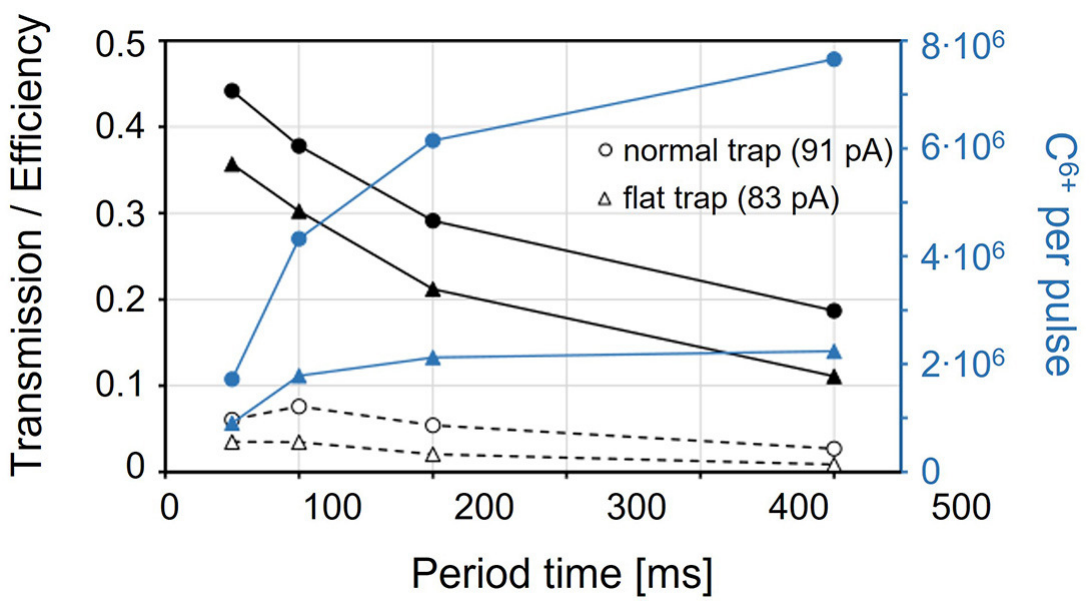
Transmission through REXTRAP (black solid line, including exiting C^+^, O^+^, H_2_O^+^ and CO^+^ beams) and total efficiency of carbon ions (charge bred to C^6+^) through REXTRAP and REXEBIS (dashed) for a normal (circle) and a flat (triangle) axial trapping potential in the Penning trap, with an input beam of 91 pA and 83 pA, respectively. The blue curves correspond to the total number of C^6+^ ions extracted from the charge breeder system (95).

Only the beam species that have been sufficiently cooled can be injected into the EBIS efficiently. Thus, in order to correctly compare the efficiency of the beam preparation inside the Penning trap for the two trap configurations, the beam has to be taken through the EBIS. The overall efficiency (dashed curves in Figure 8) of the charge breeder system for C^6+^, including REXTRAP and REXEBIS, has an optimum around 100 ms period time and is higher for the normal than for the flat trap configuration. For shorter period times, the breeding time in the EBIS is insufficient, while for longer period times losses and space charge effects in REXTRAP become important and reduce the efficiency. With the normal trap configuration at 100 ms period time, a maximum total efficiency of 8% through REXTRAP and REXEBIS could be achieved, corresponding to 4.3 x 10^6^ extracted C^6+^ ions per bunch when injecting 91 pA of CO^+^ beam into the charge breeder system, i.e. into REXTRAP. For longer period times, higher particle numbers up to 7.7 x 10^6^ C^6+^ ions per pulse could be extracted with a trade-off in efficiency. The measurements showed similar efficiencies and particle numbers for charge states 4+, 5+ and 6+, when optimizing the breeding time in the EBIS. For the lower charge states, the optimum in efficiency is reached at a shorter period time, as a shorter breeding time is sufficient.

In conclusion, the attempt to keep the molecules intact through REXTRAP using a flat trapping potential can be discarded due to the lower over-all efficiency compared to the normal trap configuration. Furthermore, even if the normal trap configuration has a reasonable maximum efficiency of 8% for the charge breeder system, when going to long period times it decreases significantly. The efficiency decreases even further for higher beam intensities, which is addressed in the next section. Therefore, standard operation charge breeding of CO^+^ together with a low-repetition-rate synchrotron would be highly inefficient.

#### Space Charge Limitations of REXTRAP and REXEBIS

Under normal conditions at ISOLDE, space charge does not play a role as typical ion currents are small compared to the capacity of the devices. As this is not true any longer for the CO^+^ charge breeding system, where significant currents need to be handled, we have made efforts to determine the intensity limitations in REXTRAP and REXEBIS. Even though the theoretical space charge limits can be calculated [details in (95)], the practical ion holding capacity might differ.

In the REX-ISOLDE case, the Penning trap turns out to be the bottleneck: the number of charges extracted from REXEBIS can go up to 5.8 x 10^9^, while for REXTRAP only up to 7 x 10^7^. This corresponds for EBIS to a filling factor k = 25%. Higher k values can be obtained, but at the cost of efficiency.

A stronger solenoidal field of the Penning trap could increase its capacity, possibly with a factor 4 going from the present 3 T to a 6 T field. Furthermore, one has to implement a correctly working rotating wall cooling scheme, in order to reach the maximum compression of the ion cloud and approach the Brillouin limit, which is currently not the case at REXTRAP where sideband cooling is the dominating effect. State-of-the-art EBISes can have a factor 10 higher space charge capacity than REXEBIS, so there would potentially be room for improvements. The measured number of charge breed particles inside an EBIS can in principle be pushed toward the theoretical limits, at the cost of efficiency. However, as the number of ^11^CO from the production stage is limited, a significant reduction in efficiency is not acceptable. In a charge breeder setup based on this concept, aiming for a transformation of ^11^CO^+^ to ^11^C^6+^ and subsequent injection into a low-repetition-rate synchrotron, the high number of ions collected over the long period time, would make the process very inefficient.

#### Continuous Injection Into the EBIS

**Figure 9:**
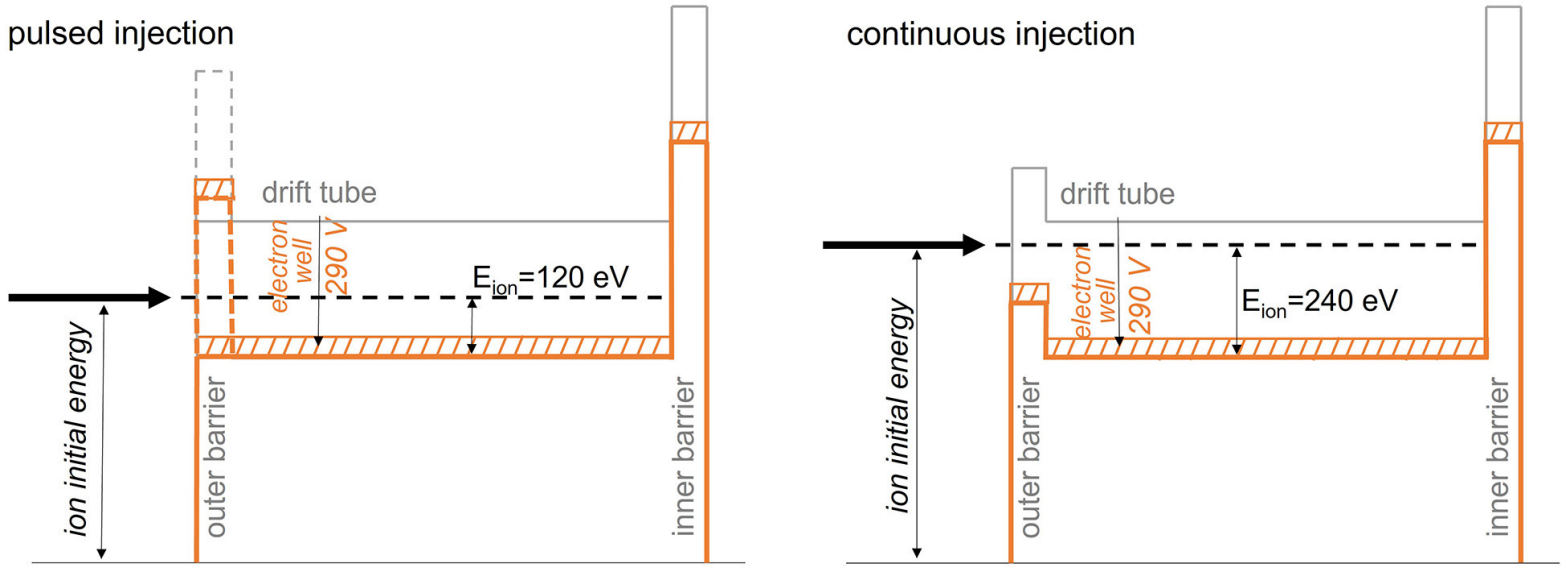
Schematic drawing of potentials for pulsed and continuous injection into the EBIS. The dashed region indicates the radial potential depth of the electron beam, in this case 40 V.

When repetition rates below 1 Hz are required, a setup with a Penning trap is not advantageous due to the high loss rate for CO and the limited space charge capacity, as discussed in section Pulsed Injection into the EBIS. Therefore, continuous ion injection into the EBIS without prior cooling and bunching in REXTRAP was tested (118). For this operational mode of the EBIS, the outer barrier of the axial trapping potential–which is usually low during the injection and high during breeding–is constantly at an intermediate voltage (see Figure 9). Ions are injected with a certain residual energy above the barrier.

During the injection, a good overlap of the ions with the electron beam is essential. If the ions are not injected fully into the electron beam, they will perform oscillations around the electrons and spend only a fraction of their time inside the beam. In the worst case, they circle the electron beam with no overlap. As the outer barrier is never completely closed, ions will escape over the barrier, unless they are ionized from 1+ to 2+ or a higher charge state by the electron beam during their first round-trip along the EBIS axial trapping potential. As this injection mechanism is in general less effective compared to the pulsed injection (119), where the ion bunch is trapped axially through the outer electrostatic barrier, a reduced trapping efficiency in the electron beam is expected. In the continuous injection mode the loss rate from boil-off of hot ions is higher compared to pulsed injection, as the energy distribution is shifted toward higher energies due to the injection conditions. In addition, the low barrier facilitates axial losses.

The CO+ beam was injected continuously over the barrier into REXEBIS during the full period time. For long period times, 6+ is certainly the most dominant charge state being extracted from the EBIS, as the charge breeding process continues during the full period time and lower charge states are over-bred.

**Figure 10:**
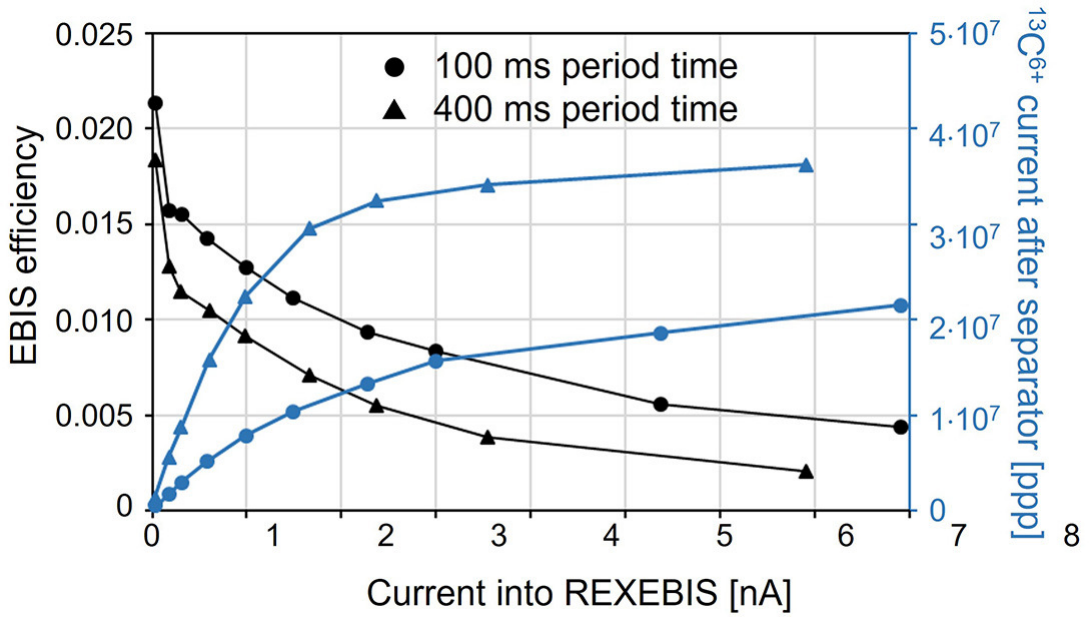
EBIS efficiency into charge state ^13^C^6+^ for an increasing input intensity injected continuously during a 100 and 400 ms period time (95).

It was found (95) that in the continuous injection mode, the EBIS cannot be filled properly as in the pulsed injection mode with a beam pulse length <30 μs. In addition, the 1+ to 6+ breeding efficiency in the order of 1% is very poor. This is summarized in Figure 10, where the period time of continuous injection is constant within one measurement series, but the injected current is increased. For 100 ms period time, the current is already saturated at a few 1 x 10^7^ C^6+^ ions extracted from the EBIS. This exemplary case corresponds to only 1% occupation of the electron beam, while residual gas ions from the EBIS itself occupies several ten percent of the space charge. One can also see that a longer injection time results in more ions being extracted, although with even lower breeding efficiency.

The low filling grade can be explained through a combination of poor injection efficiency and high loss rates through boil-off of hot ions. The breeding time to reach 6+ is longer in the continuous mode, which is a strong indication toward a poor ion-electron overlap and thereby a low trapping probability. Operation at RHIC EBIS has shown that a higher neutralization during continuous injection can be achieved when orders of magnitudes higher currents are injected, hence, at the cost of efficiency (120).

#### Conclusions on the Ion Pulse Preparation

Building an EBIS with a capacity that can in principle charge breed 1 x 10^10^ carbon ions per pulse to charge state 4+, 5+ or 6+ and extracting them in a sufficiently short pulse, is technically possible. The main challenge is to obtain a reasonable efficiency in the charge breeder system, in particular in the injection into the EBIS.

**Figure 11:**
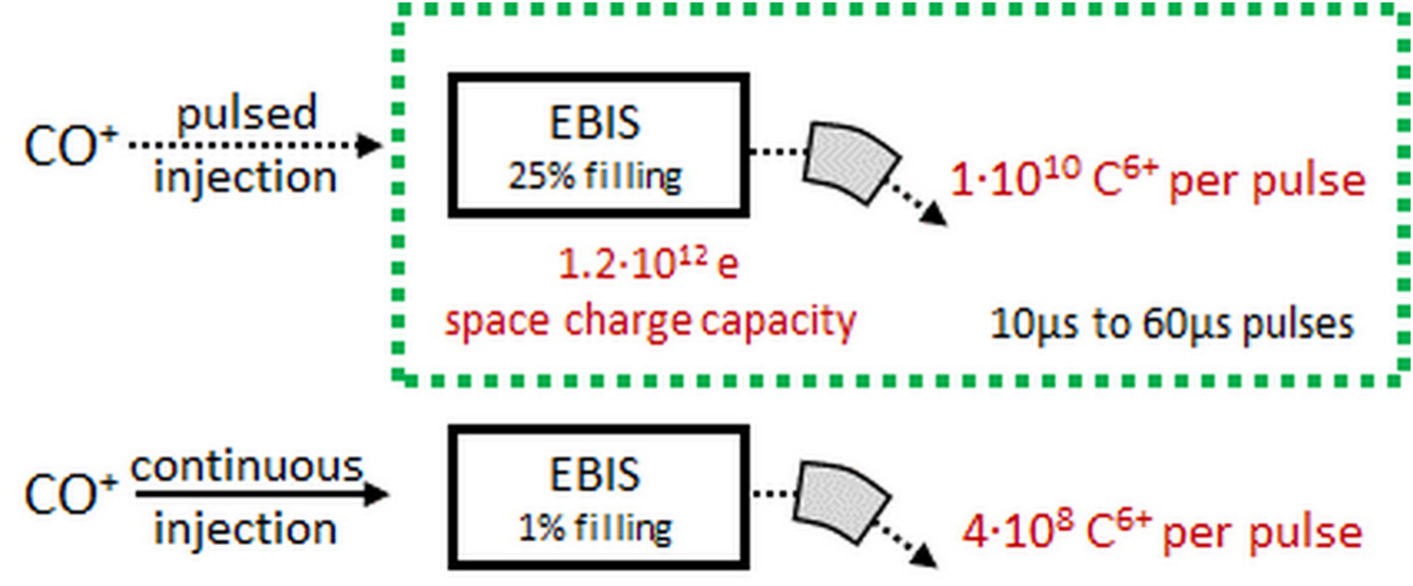
Extracted C^6+^ intensity for pulsed and continuous injection into an EBIS, assuming a space charge capacity of 1.2x10^12^ charges [adapted from (95)].

For a pulsed injection into the EBIS with a reasonable efficiency, a filling of the electron beam of approximately 25% can be reached (possibly higher if the beam is cooled before injection). To reach the desired intensity in the pulsed mode, an EBIS with an electron space charge capacity of 1.2 x 10^12^ electrons is required. This could be obtained with an EBIS of 10 A electron current, 1.8 m trapping length, and 25 keV electron energy. These specifications are similar to the RHIC EBIS (121) parameters–highly challenging, but in principle within reach with current EBIS technologies. For continuous injection, which is required when the injected pulse length is in the ms instead of μs range, the filling is significantly lower–in the order of 1%. Thus, for the continuous injection mode, an electron current sufficient to provide 1·10^10^ ions is out of technological reach. Figure 11 summarizes the two injection scenarios.

The pulse length of the extracted beam is mainly determined by the trap length and ion energy in the trapping region, as it is limited by the ion's flight time from the trapping region inside the EBIS. It does not depend on the intensity and can be as low as 10 μs, which would translate into an instantaneous current of 4 mA. By applying a ramp of a few 100 V to the drift tubes, the pulse length can be shortened further, however, it is not recommended as 10 μs is sufficiently short and the high instantaneous current would cause significant space charge effects in the low energy transfer line (122). In addition, the longitudinal energy spread might lead to chromatic aberrations in the extraction and low energy transfer systems. A short pulse length guarantees an efficient multi-turn injection into the synchrotron. Currently, at MedAustron, 50 μs pulses are used for stable carbon beams. The pulse length from the EBIS can be of the same length or shorter, thus ensuring a comparable or even improved efficiency in the multi-turn injection into the synchrotron.

For the injection into the EBIS, the following options have been considered:

- **Pulsed injection from a Penning trap**: found to be not feasible. When charge breeding CO^+^ to C^4+/6+^, the inherent problem is the low repetition rate of the synchrotron and the consequential need of storing the 1^+^ ions efficiently. As shown in section Pulsed Injection into the EBIS, the Carbon is lost in the trap with a half-life of approximately 100 ms, hence, it cannot be used to store the beam during the synchrotron cycles. In addition, the space charge limitation of the trap does not allow for efficient transmission of more than few 10^8^ ions per pulse, even when increasing the magnetic field from the present 3 T−6 T.
- **Continuous injection**: found to be unrealistic. The pulse length in the ms range of the injected beam requires a continuous injection scheme into the EBIS (in contrast to <30 μs pulses used in pulsed injection). In the tests with REXEBIS (95), we have found that efficiencies for high-intensity continuous injection are in the sub-percent range, mainly due to a highly inefficient injection and additional losses in the EBIS that prevent an efficient filling of the electron space charge potential. To reach the desired carbon intensity despite the low filling efficiency, an electron beam current significantly higher than the 1.2 x 10^12^ given in Figure 11 would be required, which is not attainable with state-of-the-art EBIS technologies. In addition, oxygen occupies more of the space charge potential of the EBIS when injecting a dioxide rather than the monoxide.
- **Pulsed injection from an RFQ cooler**: found to be limited in intensity. The capacity of the cooler may be pushed toward 1 x 10^9^–1 x 10^10^ particles, although with a large transverse emittance, resulting in a maximum of 1 x 10^9^ ^11^C^6+^ per bunch after an EBIS. However, the high intensity is challenging for the RFQ design due to the low mass of the carbon ions that requires higher frequencies than are available at state-of-the-are devices. In addition, no data on potential other loss mechanisms is presently available to the authors. The desired intensity of 1 x 10^10^ carbon ions out of the EBIS seems out of reach with this method, which is therefore only suitable if the intensity requirements can be relaxed.
- **Cryogenic trap, preferably inside the EBIS**: the preferred solution, detailed in the following.

Using a cryogenic trap allows storing the produced radioactive isotopes as neutral molecules and release them directly into the EBIS in gaseous form. A setup based on a similar concept, although with an ECR ion source, is described in (95). An advantage of neutral gas injection is that higher k values, up to >0.7, can be obtained for the EBIS. In this case, it is sufficient if the release time of the neutral molecules is in the order of some 10 ms, as the EBIS has an inherent storing capability for this time. A cryogenic trap in the vicinity of the electron beam, preferably inside the EBIS, is suggested. The neutral molecules would freeze on a cold surface [melting point CO: 68.13 K (123), CH_4_: 90.58 K (123)] and be released into the electron beam by heating of the trap. Boytsov et al. (94) have successfully demonstrated the storing of CH_4_ in such a cryogenic trap, cooled with liquid He, as well as the neutral gas injection into the electron beam of their ESIS (Electron String Ion Source) through a heating pulse of 2 ms. The conversion efficiency from frozen CH_4_ → C^4+^ of 5–10%, obtained in tests with stable ^12^CH_4_, is indeed very promising.

Coupling the cryogenic trap to an ECR ion source instead is not a valid alternative, as the ECR ion source does not have a storing capability, when operated in normal mode. The pulse length out of the source would be determined by the release time of the cryogenic trap convoluted by the effusion time to the ECR plasma and the ionization time to reach the desired charge state, which is orders of magnitudes longer than the pulse length desired for injection into the accelerator. Afterglow operation (124) provides a certain degree of storage for heavier ions, although it would need to be proven for light carbon. Furthermore, the extracted pulse length from an ECR ion source in afterglow mode is in the order of a few milliseconds and therefore too long for injection into the subsequent accelerator.

**Figure 12:**
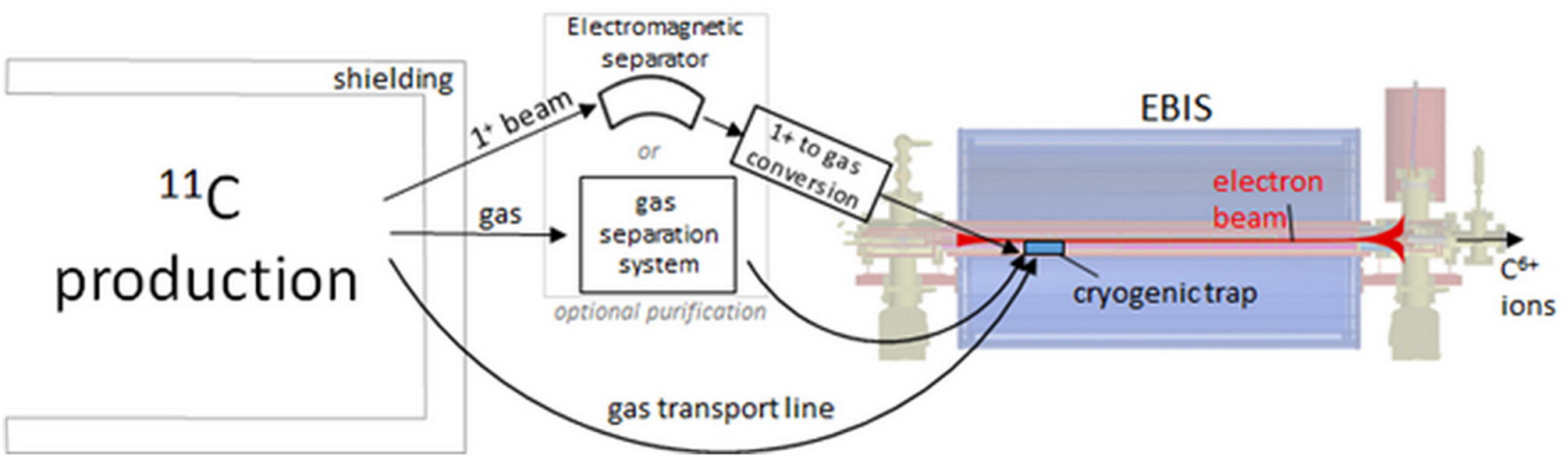
Gas injection into the EBIS, equipped with a cryogenic trap. The radioactive gas (CO or CH_4_) coming from the production target is transported into the EBIS, where it is accumulated in a cryogenic trap that releases the molecules through pulsed heating into the electron beam. If the level of contamination of other gases is too high for the EBIS, either a gas separation system or separation of a 1+ beam, in analogy to the ISOL-method, could be applied to purify the target output before injecting into the EBIS [adapted from (95)].

If the output from the target (gas or solid) is injected directly into the EBIS (see Figure 12), losses in the gas transport from the target to the cryogenic trap can be minimized by keeping transport distances as short as possible. A few meters are realistic, considering that the target area needs to be shielded. The sticking of CO to stainless steel has been found to be negligible (sojourn time 1x10^−11^ s) (125), which would result in an efficient transport. A possible complication is, that contaminations from other elements and from radiogenic ^12^C compounds that effuse from the target to the EBIS may occupy a significant fraction of the electron space charge potential. A separation of some sort is most probably required, to obtain a reasonable purity of the gas in the cryogenic trap. An approach to separate the desired gas component from contaminations is a gas separation system, as, for example, the cryogenic separation system developed by Noda et al. (88). However, it might be challenging to reach the desired purity and efficiency with such a system. Alternatively, a 1+ ion source and mass selection in an electromagnetic spectrometer, as it is done in the usual ISOL-scheme, can be considered, with a subsequent transfer and collection of the gas molecules in the cryogenic trap.

## Summary–Baseline Design and Alternatives

### The Baseline Design

The baseline design resulting from the discussions in the present chapter is the following:

- **Production stage**. Two types of targets are found as most promising: solid BN target for ISOL-type production, and gaseous N_2_ target for radiopharma-type production. Three options are compared for the driver beam: 7 MeV protons (pulsed), 18 MeV protons (continuous) and 250 MeV protons (continuous). The main criteria for selecting these options for the driver beam is the possibility of integration as upgrades to the existing therapy accelerators (using ^12^C). The choice among the presented options will depend on the operational constraints of the facility which will implement the design: batch operation or continuous production, required redundancy, space limitations, regulatory constraints.
- **Accumulation and charge breeding**. The EBIS type of ion source is selected because it presents bunching capabilities and a reasonable efficiency of CO ionization, from neutral gas to the 6+ charge state. For the stage of preparation and transport to the EBIS ion source, three scenarios have been investigated: (a) direct gas injection into the EBIS, (b) chromatography gas separation (CGS) before gas injection into the EBIS, (c) ISOL-type separation of 1+ beams, followed by deceleration to a cryotrap material inside the EBIS. The third option seems to be the best choice. The first option is discarded due to the high amount of contaminants coming from the target. The second option leads to relatively low efficiencies (when using a CO sideband, the CGS separation is difficult; when using a CO_2_ sideband, the EBIS efficiency will drop compared to the results presented for CO).
- **Acceleration**. For the acceleration, the baseline is the synchrotron with multi-turn injection, as operated at the existing ^12^C facilities. To eventually improve the typical efficiencies, shorter injection pulses can be considered.

### Alternatives for Acceleration

The presented baseline for ^11^C acceleration is centered around the design of the existing treatment facilities: synchrotron with multi-turn injection. The motivation for this was to allow the implementation of the ^11^C beams as an upgrade of existing facilities. For a green-field facility, two main alternatives can be considered:

- **Multi-pulse injection into a synchrotron**. In a state-of-the-art medical synchrotron for carbon ion therapy, only one pulse can be accepted per synchrotron fill. In multi-turn injection, the transverse phase space is completely filled after injection of one pulse, as the phase-space-painting covers the acceptance of the ring. Even if it was not covered immediately after the filling with one pulse, still the injection of multiple pulses from the source would not be possible due to the phase space filamentation in the ring. In a future synchrotron-based therapy accelerator, the accumulation of several pulses in the ring could be realized through electron cooling in the synchrotron (126). The cooling reduces the transverse emittance in the ring and therefore several pulses can be injected. The method is successfully applied at several storage rings, for example the ESR (Experimental Storage Ring) at GSI (127), ELENA (Extra Low ENergy Antiproton ring) (128) and LEIR (Low Energy Ion Ring) (129), the latter two at CERN. Assuming that 10 pulses can be accumulated, the intensity requirement on the EBIS would relax by a factor 10, but considering that the cooling time for each injection pulse might be in the order of 1 s, one might prefer to still keep the requirement for a high EBIS intensity and rather gain on the total spill intensity (as only a reduction of the treatment time can justify the extra complexity of the accelerator).
- **Linear acceleration**. A more natural choice for acceleration of ^11^C is a linear accelerator. In comparison to a synchrotron, it can be more easily combined with an EBIS, as both machines are inherently pulsed. Designs of Linac-based carbon ion facilities have been proposed by the TERA foundation in the form of CABOTO—Carbon Booster for Therapy in Oncology—an all-Linac accelerator for C^6+^ ions (130, 131), and by CERN within the PIMMS2 study (132). The repetition rate for the former may be as high as 400 Hz and the beam energy can be changed between pulses by switching on or off the cavities as required. This allows for fast spot scanning of the tumor, which could also follow tumor movement caused by the patient breathing. In substituting the synchrotron with a Linac in our ^11^C acceleration scheme, one eliminates the two major problems: the high required per-pulse-intensity and the storing of the produced radioactive isotopes, either as molecules or as ions. The primary source concept for stable carbon in the CABOTO design is an EBIS equipped with MEDeGUN (133, 134), a high-compression electron gun, developed at CERN. MEDeGUN is designed to provide >1 x 10^8^ C^6+^ ions per pulse at 400 Hz from ^12^CH_4_ gas. According to the calculation in (133), which includes gas transport from outside the EBIS to the ionization region, 9.2 x 10^−7^ mbar·l/s or 3 x 10^12^ CH_4_ molecules per second need to be provided to the gas supply line in order to reach the desired ion intensity. If this system was to be used for radioactive beam, a gas purification system might be required, as discussed above. The repetition rate of 400 Hz requires charge breeding to 6+ in under 2.5 ms, which can only be realized in a high-density electron beam. Therefore, the main focus of MEDeGUN is on the high compression of the electron beam, rather than a high capacity. Compared to the RHIC-like EBIS discussed in section Ion Pulse Preparation with an 800 μm electron beam radius, the MEDeGUN beam is highly compressed down to a radius of 60 μm. The small electron beam radius also helps keeping the emittance low, which is beneficial for the design of the consecutive Linac. As a drawback, however, the ion injection acceptance is also relatively small, which would complicate a 1+ injection. In our case, however, gas from the target would be injected continuously, thereby eliminating the need for storing the produced radioisotopes.

### Alternatives for the Injector

Most of the complexity of the baseline design stems from two constraints:

1. The need of **accumulating the** ^**11**^**C particles**, due to the limited amount that can be created in continuous mode by using a target.
2. The need to create **high charge states** (≥4+), due to the constraints of the linear accelerator of the injector in the existing facilities (operating for a ratio Q/M ≥ 1/3). The option of beam stripping is only efficient at beam energies of at least a few MeV/u (3), so for the existing designs an additional loss of efficiency has to be considered if the stripping is done at lower energies.

The constraint (1) is strengthened by the constraint (2), which requires a 2-stage ionization and thus decreasing the overall efficiency of converting the ^11^C atoms produced in the target to the final high-charge state ions. If the constraint (2) is relaxed, for example by using a Linac able to accelerate ions with lower Q/M ratios, then a simplified ionization scheme becomes possible: a single ion source can be used, coupled as closely as possible to the production target. As this is the general description of the ISOL ion sources, the natural place to look for solutions are the ISOL ion sources. In this case, the elimination of the contaminants can to a significant extent be done within the same compact unit, between the target and the ion source.

Charge states of 1+ or 2+ are achievable by several ISOL ion sources, of arc discharge type or of ECR type. Interesting results have been obtained recently with a pulsed operation of a VADIS ion source (116). An ionization efficiency of 30% for ^12^C^+^ is reported for generating a pulse of 100 μs after an accumulation time of 1 ms (only within the ion source volume); if increasing the accumulation time to 100 ms, the efficiency is still of 6%. If a BN target is used, Table 4 shows that approximately 5 x 10^13^ molecules of ^11^CO_2_ are expected to come out of the target, per second. To get out of the ion source an intensity of 5 x 10^9^ of ^11^C^1+^ per second, a conversion rate to atomic ^11^C (from the molecular CO_2_) of ≥0.01% is needed. That is considered achievable considering the typical spectrum of ions coming out of a VADIS ion source, but further investigations are needed for validating this option, end-to-end. The VADIS ion source can even deliver 2+ ions, and the efficiency of producing C^2+^ from an input gas of CO_2_ is also a subject of these further investigations.

## Integration to Existing Facilities

The possible integration to existing facilities is analyzed for one specific case: MedAustron. This analysis is considered to be applicable for all other synchrotron-based facilities based on the PIMMS design.

### General Facility Description

**Figure 13:**
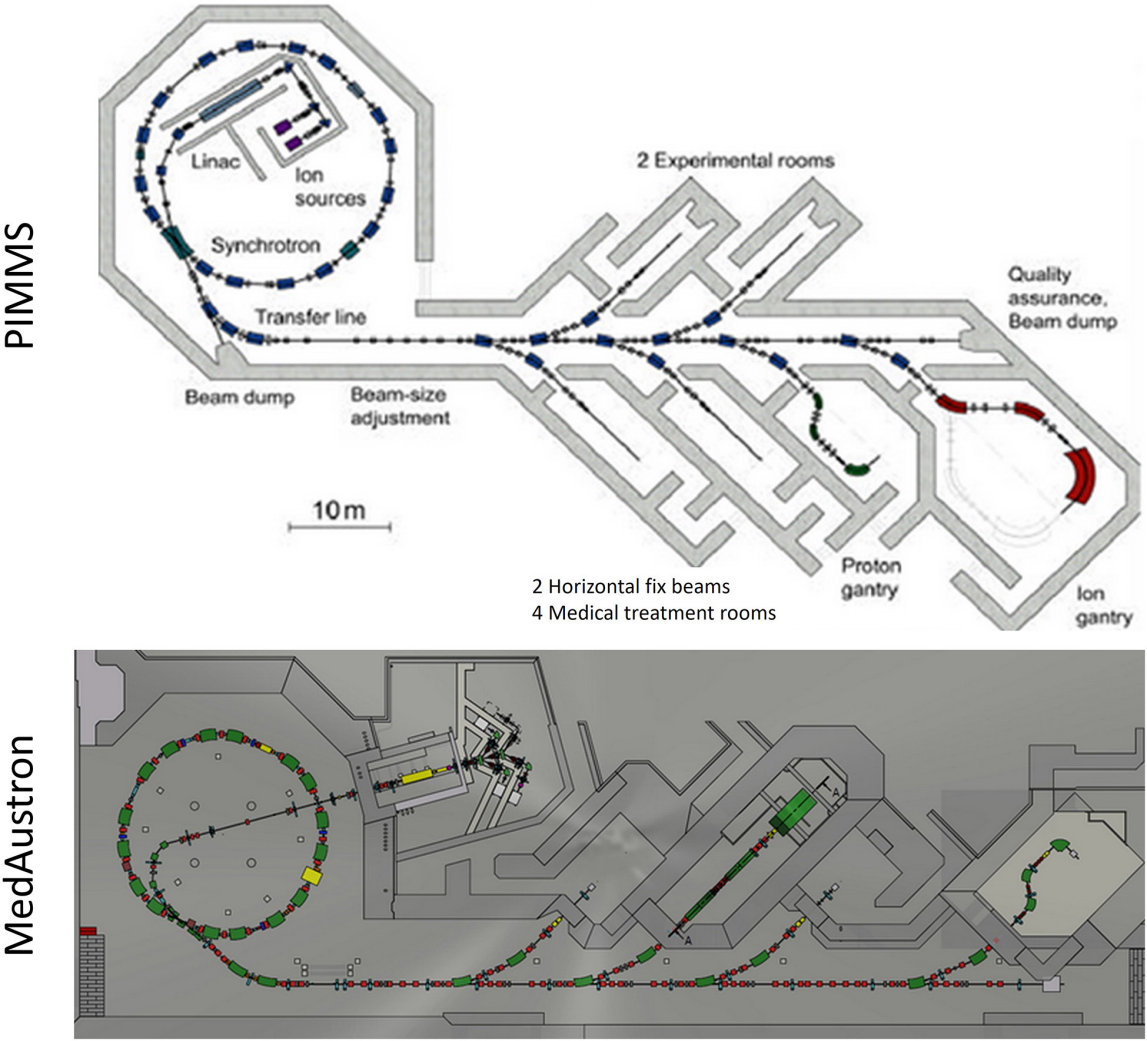
**Top**: A potential layout of a carbon/proton treatment facility as proposed in the PIMMS study. **Bottom**: Actual MedAustron facility layout: a separated injector hall which houses 3 ECR ion source and potentially 5 source branches, completely enclosed Linac bunker for RFQ and IH-Tank, Synchrotron hall and the long extraction line which distributes the beam to Irradiation Rooms 1–4.

MedAustron is a synchrotron based multi room treatment facility whose design was derived from CERN's PIMMS (3) study (see Figure 13, upper part). This study foresees two ECR ion sources for proton and carbon ion beam production feeding a Linac for acceleration up to 7 MeV/u. *Via* a medium energy beam transfer line (MEBT) the beam pulse is injected into a 77 m circumference synchrotron where it is bunched and accelerated to the desired energy. A third order resonant extraction scheme is applied to extract the beam for 0.1 to 120 s spills. A high energy beam transfer line (HEBT) distributes the spill to the requested irradiation room. Each room is equipped with a dose delivery system for active pencil beam scanning.

Although both CNAO and MedAustron are based on this very same study there exist several peculiarities of each center. Both synchrotrons are based on the same lattice design, the few differences in magnet design to improve ramping behavior and field homogeneity as well as better suppress eddy currents may be neglected in this report. The main distinctions between the two facilities lie in the injector setup and the HEBT concept.

While the PIMMS design foresees a short MEBT with Linac and sources within the synchrotron ring, the MedAustron design (Figure 13, lower part) utilizes a long MEBT which crosses the synchrotron ring to place both Linac and sources outside of the synchrotron ring. As all high-power RF structures (RFQ, IH-Tank) are contained within a bunker the injector hall remains accessible during operation which facilitates service and installation actions. A Low Energy Beam Transfer line (LEBT) feeds the RFQ of the Linac and enables the user to select ${\text{H}}_{3}^{+}$ or ^12^C^4+^ beams from different ion sources *via* two switching dipoles which are connected to 5 potential source branches. Currently 3 source branches are fully installed leaving space for future source developments.

Concerning the HEBT realization MedAustron very much follows the original PIMMS design. The magnetic septa for synchrotron extraction are followed by a dispersion suppressor bend to reduce dispersive effects. Within this bend fast chopper magnets steer the beam around a chopper dump whenever the beam is requested by a medical safety system. The first part of the straight HEBT section is a phase stepper and phase shifter (PSS) which is used to adapt horizontal and vertical beam sizes *via* rotation of the bar of charge/emittance ellipse in the horizontal/vertical phase space respectively. Beam size and symmetry is supposed to be set here relying on non-manipulative transport of these properties along the rest of the HEBT which is realized by straight telescope modules and double bend achromat optics in the bend tuned to the final part of the corresponding transfer line.

### Potential ^11^C Scenarios

#### Linac Production

**Figure 14:**
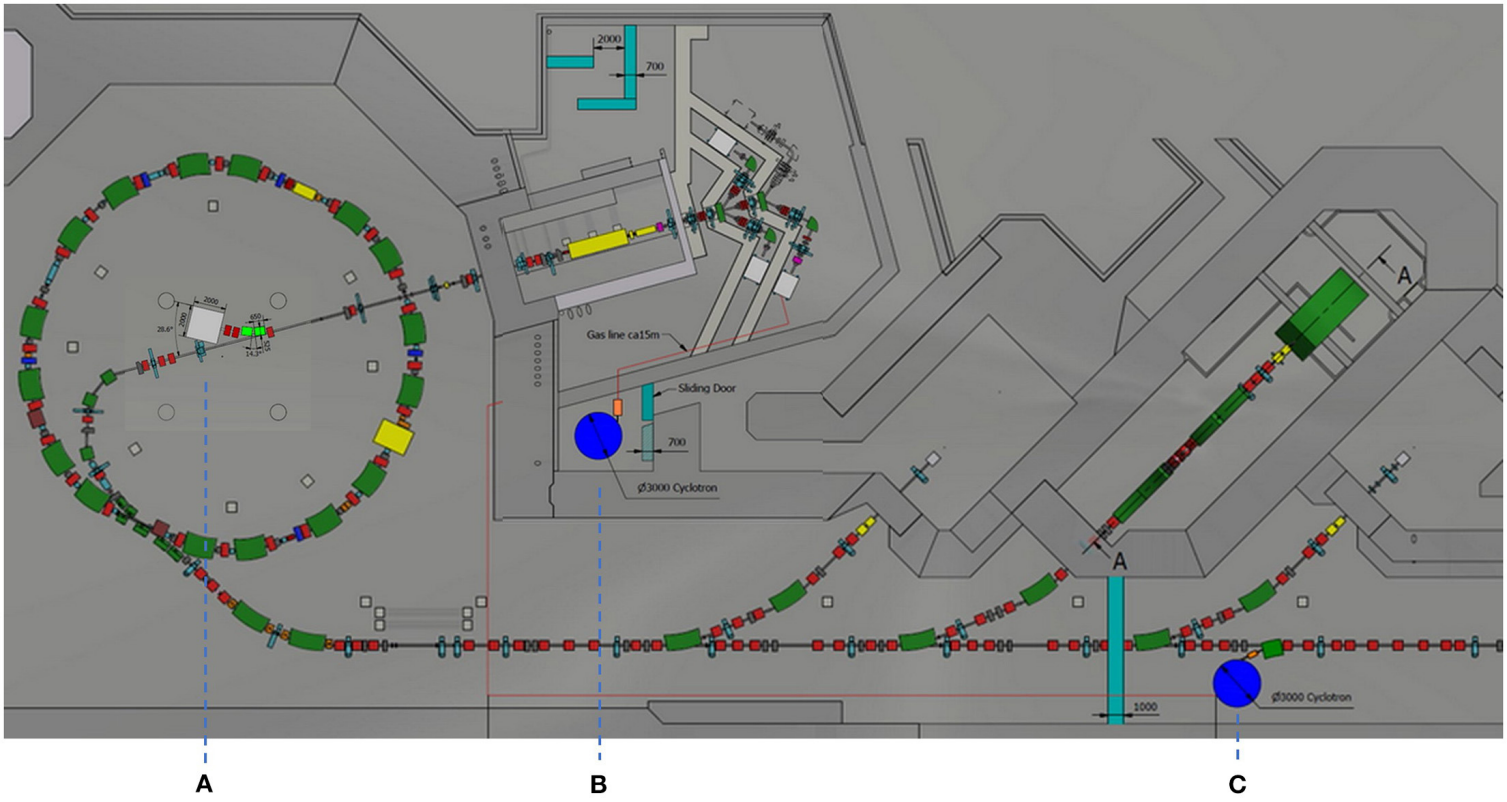
**(A)** Potential production facility layout in the existing Linac: a new fast deflector deviates the beam sufficiently to hit two magnetic septa (green) which steer the beam toward an activation target housed in a concrete shielding. **(B)** Medical Cyclotron (blue) option to enable parallel beam operation with protons on the Gantry, additional concrete walls (turquois) in the extraction line and at entrance of the injector hall. The production target is connected to the Injector hall *via* a long gas feed line. **(C)** Radiopharma Cyclotron (blue) solution implemented in the existing cool down area of IR1 which should also house the production target (orange). Additional radiation protection doors and mace will have to be installed to avoid new access restrictions in near installations.

A very convenient upgrade scenario in the sense of installation costs would be to use the existing Linac (135) for ^11^C production. A potential layout is depicted in Figure 14A which features an electrostatic deflector to achieve kicks of up to 10 mrad which sufficiently displaces the beam 9 m downstream of the MEBT (136) to hit a series of two magnetic septa with a bending angle of 14.5° each. The target station is supposed to be housed within a 70 cm thick concrete structure to keep surrounding radiation levels below 13 μSv/h. This will ensure no major impact on service intervals and spontaneous interventions.

To achieve acceptable activation levels of a potential ^11^C source sufficient beam current must be provided. The MedAustron Linac is a pulsed structure with a 10 Hz repetition rate and typical rf pulse lengths of 400 μs. Beam transport typically only covers 30–50 μs of such a Linac pulse. In addition, only one pulse out of ca. Seven seconds is actually used for beam transport. Yet the RF structures, consisting of a RFQ, Buncher Cavity, IH-Tank and a Debuncher Cavity keep pulsing at 10Hz to maintain thermal stability. Therefore in 98.5% of operation time the Linac is not occupied by beam.

Typical beam currents during a beam pulse can reach up to 800 μA (137), which means that if the full 400 μs of each 10 Hz pulse is used for beam transport it results in 3.2 μA DC current equivalent. Potentially the duty factor of the Linac could be further increased if the thermal load is properly taken into account. In addition, ion source optimization in terms of beam current offers additional potential, seen that beam property constraints for the synchrotron injection do not need to be respected any longer (bottleneck will be RFQ and IH-Tank transmissions). In total a current boost to 5–7 μA DC is expected without major implications.

Assuming a BN target (99) and a 7 MeV beam the expected saturation yield for ^11^C production is around 1.7 GBq/μA which would add up to 12 GBq (for ^11^C only). This is on the lower end of the spectrum needed for a functioning facility and would produce tight requirements on the performance and efficiency of other components in the production line. Alternatively, a proton ion source upgrade could close the gap and boost beam intensities by another order of magnitude. To remain compatible with clinical safety restrictions such a source would need to be very stable and reproducible. In addition, a flexible intensity reduction method would be needed to stay within clinical intensity ranges (138). This can be either a systematic intensity reduction downstream of the activation target, at synchrotron injection, or a transverse beam blow up at RFQ entry. For the latter method, a suitable quadrupole could be adopted similar to the intensity reduction schemes already in place at Heidelberg (HIT).

#### Medical Cyclotron

Although the MedAustron facility runs both protons and carbons there is one beam line which will be exclusively used with proton beams. The Gantry in IR4 is based on Gantry2 by PSI and only supports low Bρ beam rigidities which are not suitable for carbon. Therefore, it is proposed to install the medical cyclotron in the HEBT which feeds IR4 exclusively which will enable parallel beam operation in two rooms, thus increasing redundancy and offering a backup treatment room in case of unexpected downtime (Proton treatment plans for carbon patients would be needed as backup). Assuming a treatment duration of 15 mins and 10 mins patient preparation time (in room) the parallelization of treatments would result in an increase of the yearly applied fractions by 40%. Not only is the yearly patient throughput affected but also the quality of the machines can be improved as the Synchrotron facility and the connected transfer lines can be optimized for a different Bρ range (139). This would offer the option for IR1, IR2 and IR3 to run with ^12^C^4+^ carbon and ${\text{H}}_{3}^{+}$, which require an almost identical Bρ range of 3.1-6.78 Tm, while the gantry is solely used with Protons. The introduction of stripping foils upstream of the bending to each room provides ^12^C^6+^ and H^+^ in the irradiation rooms. Reducing the beam rigidity span of extracted beams is advantageous to improve the field-quality. If the accelerator is used in a wide range of nominal fields (e.g., 1.1–6.78 Tm), especially at high field-levels, the field distribution will be different from the distribution at low field-levels because of saturation effects. For this reason, the field distribution optimized at low field-levels will not be satisfactory at high field-levels and vice versa. From this point of view, shrinking the beam rigidity interval is beneficial. In addition, the magnet power supplies will be operated in a reduced interval of nominal currents. The “low-Bρ” region, i.e., the low nominal currents, is removed. This is expected to improve stability and to reduce ripple during extraction flat-tops. Better stability and lower ripple have a smoothing effect on the spill time-structure, hence improving the spill quality.

As indicated in Figure 14B, the medical cyclotron could be positioned in the T4 beamline before the rotator, which would no longer be needed to remain movable and could serve as a static matching section from the cyclotron to the existing beam line. Between the Cyclotron and rotator a switching dipole shall be introduced to enable the option of feeding IR4 using the synchrotron further increasing redundancy within the facility. Under the assumption that radiation protection walls are properly positioned between the transfer lines and the cyclotron, service and maintenance tasks may be executed on one machine while operating the other which will result in more regular service slots, increased preventive maintenance and a reduction of down time.

Medical cyclotrons which are used for treatments are available off the shelf (140) and can provide proton energies up to 250 MeV with typical currents <1μA (e.g., Varian ProBeam). Yet a cyclotron for radioisotope production must provide higher currents then typically required for medical treatments. Thus it is proposed to either install multiple ion sources in the cyclotron providing different particle fluxes to enable a high and a low current mode or chose a different cyclotron option like the high intensity superconducting cyclotron (HISCC) (141) proposed by the Massachusetts Institute of Technology. HISCC is designed to deliver proton beams up to an energy of 250 MeV for currents up to 1 mA while maintaining a maximum loss extraction rate of 0.1%. For either option a basic requirement would be that the output intensity can be adapted rather quick to drive a radioisotope station in between treatments or beam requests from the irradiation room. As cyclotrons usually have multiple beam extraction ports parallel operation of treatment and Isotope production is possible. Yet the beam currents during treatment mode will be reduced which means that during parallel operation no more than a mere sustainment of the activity in the target material may be achieved.

Overall a medical cyclotron would increase the performance and annual turnover of the treatment center as a whole and improve machine redundancy while reducing down time. Yet the high proton energy results in the need of a different target design the implications of which remain to be studied to give a detailed assessment on production efficiency. In addition, radioisotope production with a 250 MeV primary beam increases the number of different isotopes produced vastly and will thus require an efficient mass separation method. Thus a CO^+^ source and electromagnetic mass separation might have to be used. Transport to the injector hall could either be established *via* a CO^+^ beam line, which would further increase installation costs, or *via* a long gas pipeline which would affect transport efficiency due to the long transit time in comparison to half-life. The integration of an additional therapy cyclotron calls for more detailed simulations to study and fine tune the compatibility with the existing high energy transfer line and gantry.

#### Radiopharma Cyclotron

Potentially the best option for ^11^C production is to introduce a commercially available radiopharma cyclotron. Such machines are typically designed to deliver proton energies of 15 to 20 MeV at currents between 100 and 300 μA available on up to eight extraction ports [e.g., IBA Kiube (142)]. Off-the-shelf cyclotrons of that energy range are very compact in size, typically 2 x 2 m, which facilitates integration into an existing infrastructure. In the case of MedAustron it could easily be placed in the injector hall or even in a dedicated radioactive cool down chamber (see Figure 14C) which would separate the cyclotron and the activation target from the rest of the ^11^C production facility, leaving it accessible for maintenance. Expected total activity of a potential activation target would be 10–12 Sv/h which comprises many different radioisotopes produced. If needed a chromatography gas separation system or another mass separation system could be installed in the injector hall. A new EBIS with a cryotrap for further purification could replace Source 3 and use this branch for beam insertion into the existing facility. Only minor modifications would be needed to the injector hall to not compromise the existing radiation protection strategy e.g., a maze at the entry of the injector hall. Access during operation will be restricted but due to the short half-life not persistent.

A radiopharma cyclotron is the best trade-off between installation costs and production facility performance. Depending on the chosen option and internal shielding possibilities acquisition costs of EUR 700 k to 1,100 k for a commercially available cyclotron can be expected.

### Constraints

#### Legal and RP Constraints

Austrian law foresees strong interactions with the authorities. Particle accelerator facilities which provide kinetic energies of more than 50 MeV must undergo an Environmental Impact Assessment (EIA) to propose and proof a concept of minimizing any impact on the surrounding public. This process is typically started years before beginning construction and implies a detailed evaluation of appointed experts by the authorities. After several iterations it typically results in a provisionary operation authorization with regular measurements and survey reports. A final verification measurement which proofs the proposed concept to fulfill its initial assumptions and calculations closes the EIA process. For minor changes a processing time of 1.5–2 years can be assumed. The MedAustron EIA for the overall facility was first filed in October 2009 and resulted in a positive decision by the authorities in December 2010, yet the final closure is envisaged in 2022 with the finalization of the gantry. Facilities which employ accelerators below 20 MeV are not required to perform an EIA evaluation, but it suffices to apply for an operation authorization under the Austrian radiation protection law. The same relaxation of laws is applicable for radiopharmaceutical cyclotrons e.g., in hospitals.

Radiation protection limits which must be respected, for a scenario where no restriction on duration of stay within the respective zones are issued, are:

- 0.5 μSv/h in Public Area (Outside of Building)
- 3 μSv/h in Supervised Area
- 15μSv/h in Controlled Area.

If access limitations to the MedAustron premises (parking and open space outside of building) would be established, the public RP limitations would only be applicable outside the MedAustron premises. In addition, the supervisory area in the corridor which connects the synchrotron entry door with the IR1 access door could be elevated to become a controlled area. If the given dose rates for supervisory and control areas cannot be respected, additional access limitations concerning the duration of stay within the respective areas must be implemented. Such systems are not foreseen at MedAustron so far.

Assuming an activity of target material of 1.6 Sv/h additional RP measures must be implemented. In a first approximation a 70 cm concrete shielding should be sufficient to reduce the dose rate below controlled area limits. In the case of a 20 MeV cyclotron installation in the existing cooldown chamber it would result in an additional RP door to remain accessible during operation. A medical cyclotron installation in the HEBT would need an RP wall to close off the gantry transfer line. Should the existing Linac be used the respective shielding walls shall be introduced around the activation target in the synchrotron hall.

#### Operational Constraints

A primary goal of any potential installation is to keep the impact on daily operation and regular maintenance tasks to a minimum. Ideally both ^11^C operation and maintenance will be possible in parallel (see above scenarios). Implementation of previously described radiation protection measures (143) in the synchrotron hall, the extraction line or the cool down chamber ensures the independent maintainability for synchrotron, medical cyclotron and/or radiopharma cyclotron. For all the presented options an increased level of radiation is to be expected in the vicinity of the ion sources. Therefore, access restrictions to the injector hall must be implemented during, and for a certain cool down time after, ^11^C production runs. An ambient air activity monitoring on the exhaust of the ventilation system is already in place. This could serve as a conditional measure to authorize access after ^11^C production runs but also to ensure minimum impact on the surrounding environment. Additionally, RP measures in the form of an entry mace at the injector hall doorway will have to be instated.

### Potential Timeline

**Figure 15:**
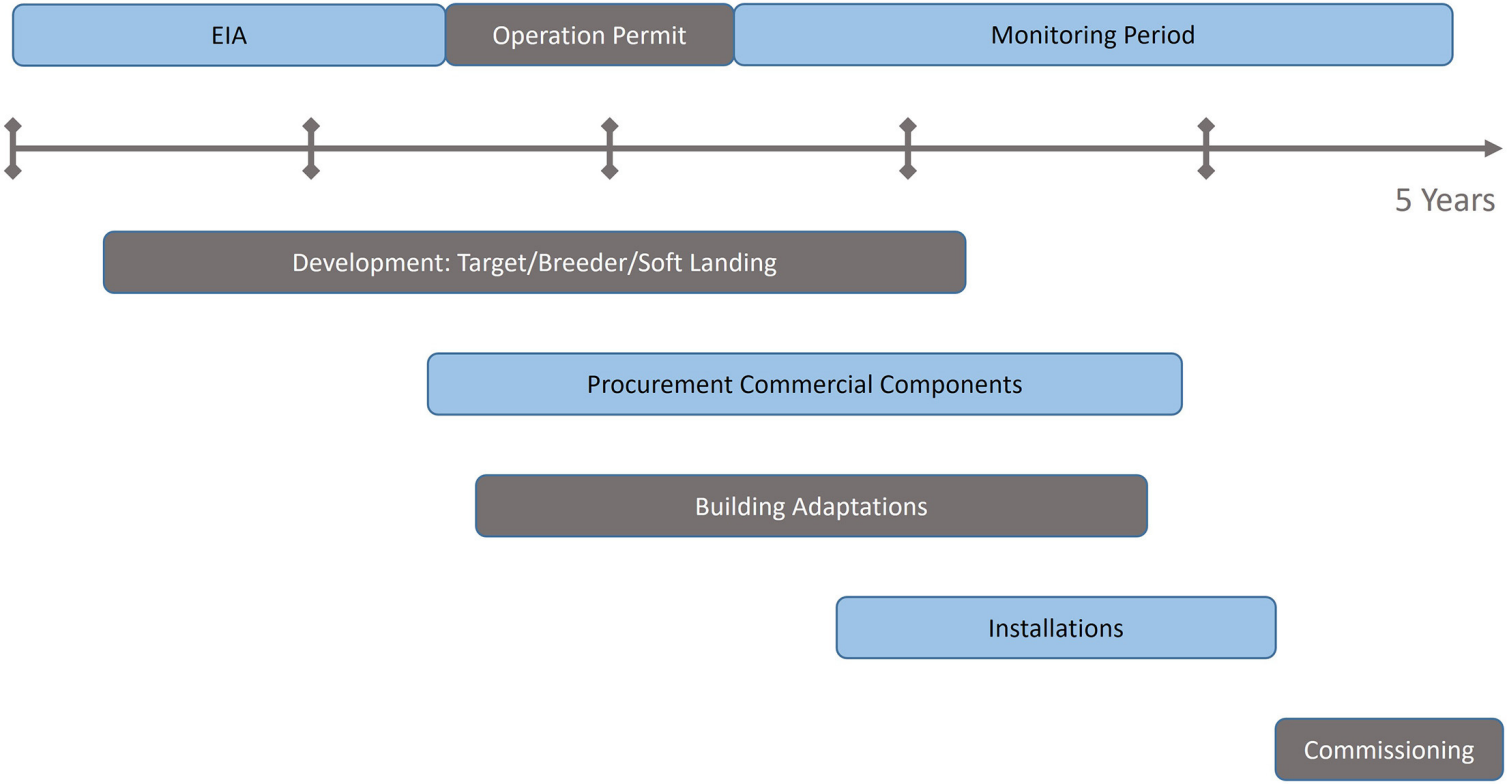
Potential integration timeline under the view of the constraints at MedAustron taking actual development status and legal constraints into account.

The first step toward a successful integration of a ^11^C production facility in an existing hadron treatment center is to commence the EIA and negotiations with authorities on provisional operation permit conditions. It is estimated that the approval of the EIA concept can be achieved within 1.5 years which will result in a provisionary operation permit and an according monitoring period including regular reports to authorities. With initial EIA approval construction on building adaptations may begin. In parallel procurement of commercially available components shall be triggered. Any developments required for the online production line shall be triggered as early as possible and are estimated to be finalized within 3 years (as indicated in Figure 15). Installations shall start as soon as the local construction site permits it. Approximatively 4 years after project kick-off the commissioning period shall be started. First radioactive beams are expected in clinical trials within 1 year after beginning of commissioning which results in a total timeline of around 5 years. If development of crucial components can be triggered beforehand the implementation time may be significantly reduced.

## Conclusion

In this manuscript, we have reviewed the different aspects important for the production and acceleration of ^11^C at required intensities to be used with imaging PET scanners at an existing treatment facility. The production of ^11^C beams for patient treatment and quality assurance of the delivered beam is challenging, but technically possible. Different production routes can be envisaged, the solution that provides the highest margin includes the implementation of a dedicated cyclotron suited for radiopharmaceuticals, exploiting either a gas target or a solid ISOL BN target. The first post acceleration stage requires using a charge-breeder with a cryotrap directly fitted in the EBIS ion source, to avoid the bottleneck of space charge limitation of the EBIS filling. In this case, impurities are not believed to significantly saturate the ion source and will be separated away from the post accelerated ^11^C ions. Following these estimations, a baseline design is proposed, as well as alternatives for acceleration and injector components. A transition toward the next-generation treatment facilities can be done *via* the upgrade of existing facilities, which is detailed by taking the example of the MedAustron facility.

## Data Availability Statement

The raw data supporting the conclusions of this article will be made available by the authors, without undue reservation.

## Author Contributions

LP: sections Required Accelerator Layout and Summary-Baseline Design and Alternatives and general document setup and review. CS: section Integration to Existing Facilities. RA and EF: section Motivation for Carbon-11 Beams: Overview and Modeling. JP: section Ion Pulse Preparation. SS: sections Production of Carbon-11 Beams: Overview of Past Results and Radioisotope Production. TC, FW, and TS: review and contributions to all sections. KP and AF: review and contributions to section Motivation for Carbon-11 Beams: Overview and Modeling. All authors contributed to the article and approved the submitted version.

## Funding

The MEDICIS-Promed project has received funding from the European Union's Horizon 2020 Research and Innovation Programme under grant agreement No. 642889.

## Conflict of Interest

CS is still employed by MedAustron. LP was employed by MedAustron until 2016. The remaining authors declare that the research was conducted in the absence of any commercial or financial relationships that could be construed as a potential conflict of interest.

## Publisher's Note

All claims expressed in this article are solely those of the authors and do not necessarily represent those of their affiliated organizations, or those of the publisher, the editors and the reviewers. Any product that may be evaluated in this article, or claim that may be made by its manufacturer, is not guaranteed or endorsed by the publisher.
